# The glymphatic system for neurosurgeons: a scoping review

**DOI:** 10.1007/s10143-024-02291-6

**Published:** 2024-01-23

**Authors:** Mohammad Al Masri, Alba Corell, Isak Michaëlsson, Asgeir S. Jakola, Thomas Skoglund

**Affiliations:** 1 Department of Clinical Neuroscience, Institute of Neuroscience and Physiology, Sahlgrenska Academy, University of Gothenburg, Gothenburg, Sweden; 2 Department of Neurosurgery, Sahlgrenska University Hospital, Blå Stråket 5, 3 tr, SE-41345 Gothenburg, Sweden

**Keywords:** Glymphatic system, Neurosurgery, Brain tumors, Hydrocephalus, Head injuries, Aquaporin-4

## Abstract

The discovery of the glymphatic system has revolutionized our understanding of cerebrospinal fluid (CSF) circulation and interstitial waste clearance in the brain. This scoping review aims to synthesize the current literature on the glymphatic system’s role in neurosurgical conditions and its potential as a therapeutic target. We conducted a comprehensive search in PubMed and Scopus databases for studies published between January 1, 2012, and October 31, 2023. Studies were selected based on their relevance to neurosurgical conditions and glymphatic function, with both animal and human studies included. Data extraction focused on the methods for quantifying glymphatic function and the main results. A total of 67 articles were included, covering conditions such as idiopathic normal pressure hydrocephalus (iNPH), idiopathic intracranial hypertension (IIH), subarachnoid hemorrhage (SAH), stroke, intracranial tumors, and traumatic brain injury (TBI). Significant glymphatic dysregulation was noted in iNPH and IIH, with evidence of impaired CSF dynamics and delayed clearance. SAH studies indicated glymphatic dysfunction with the potential therapeutic effects of nimodipine and tissue plasminogen activator. In stroke, alterations in glymphatic activity correlated with the extent of edema and neurological recovery. TBI studies highlighted the role of the glymphatic system in post-injury cognitive outcomes. Results indicate that the regulation of aquaporin-4 (AQP4) channels is a critical target for therapeutic intervention. The glymphatic system plays a critical role in the pathophysiology of various neurosurgical conditions, influencing brain edema and CSF dynamics. Targeting the regulation of AQP4 channels presents as a significant therapeutic strategy. Although promising, the translation of these findings into clinical practice requires further human studies. Future research should focus on establishing non-invasive biomarkers for glymphatic function and exploring the long-term effects of glymphatic dysfunction.

**Supplementary Information:**

The online version contains supplementary material available at 10.1007/s10143-024-02291-6.

## Introduction

The lack of a lymphatic system in the central nervous system (CNS) has long intrigued scientists. In 1914, Lewis H. Weed published a study that outlined an alternative drainage pathway for cerebrospinal fluid (CSF) [1]. By injecting Prussian blue, a blue pigment, into the subarachnoid space, he found pigment deposits in cervical lymph nodes and lymph channels. This demonstrated that CSF drains into the lymphatic system. However, subsequent studies struggled to identify the mechanism for clearing interstitial solutes and metabolic waste from the brain interstitium until just over a decade ago.

In 2012, Maiken Nedergaard with colleagues published a paper introducing the concept of the glymphatic system, necessitating a revision in our understanding of the circulation of CSF within the CNS [2]. Serving as a brain-wide drainage pathway, the glymphatic system facilitates the removal of waste products from the brain by managing the circulation of CSF throughout it. From the subarachnoid space, CSF traverses through the perivascular spaces (PVS, also known as Virchow-Robin spaces) into the brain’s interstitial space, a flow modulated by aquaporin-4 (AQP4) water channels in astrocytes [3]. This fluid is subsequently drained into the perivenous spaces and other conduits, eventually reaching the meningeal lymphatic system, and culminating its journey in the deep cervical lymph nodes [4]. The recent finding of a fourth meningeal layer, the subarachnoid lymphatic-like membrane, has added even more complexity to the new model of CSF dynamics [5]. Importantly, sleep has been shown to optimize glymphatic clearance, with possible implications for neurodegenerative disease processes [6].

Much is still unclear, but the body of knowledge in this area of research is growing rapidly. Early studies were based on animal experiments, but it has now been demonstrated that the glymphatic system exists in humans and that it can be visualized and quantified using MRI technology [2, 3]. Several studies also point to the role of the glymphatic system in various disease processes in the brain [7].

As our understanding of the glymphatic system grows, this scoping review aims to provide an overview of existing knowledge and explore the potential involvement of the glymphatic system in various neurosurgical conditions. This includes diseases potentially resulting from dysfunction in the system, such as normal pressure hydrocephalus and idiopathic intracranial hypertension (IIH). In other settings, like subarachnoid hemorrhage (SAH) and traumatic brain injury (TBI), the disease itself may trigger dysfunction of the glymphatic system and consequently impact the course of the disease.

## Materials and methods

### Search strategy and study selection

To identify relevant articles, a search from January 1, 2012, to September 30, 2022, was performed in the online databases PubMed and Scopus. In PubMed, the search term “glymph*” (used as a wildcard to cover variations such as “glymphatic”) was used in “All fields,” while in Scopus, the search was conducted using the term “glymph*” in the “Title,” “Abstract,” or “Keywords” fields. The search was subsequently updated on October 31, 2023, to ensure the most recent content was included. The search results were exported into Excel, and duplicates were removed.

In the initial screening, the titles and abstracts of the articles were sequentially reviewed by two authors (TS and MAM) and, in cases of doubt, discussed with a third review author (ASJ). Eligible articles were peer-reviewed original studies, had full-text available, and were deemed to have potential neurosurgical relevance, i.e., studies with any connection to neurosurgical diseases such as normal pressure hydrocephalus, IIH, stroke, including SAH and intracerebral hemorrhage (ICH), neuro-oncological conditions, and TBI. Exclusion criteria included gray literature, non-English language articles, and review papers. Papers related to both human and animal studies were included.

### Data extraction

The selected articles were reviewed in full text and the data extraction was performed independently by two reviewers (MAM and AC). Studies that raised questions or uncertainties were discussed with a senior neurosurgical consultant (ASJ). Since we anticipated collecting heterogeneous and limited data, the study was designed to be purely descriptive, i.e., we did not plan for any statistical or meta-analysis.

**Fig. 1:**
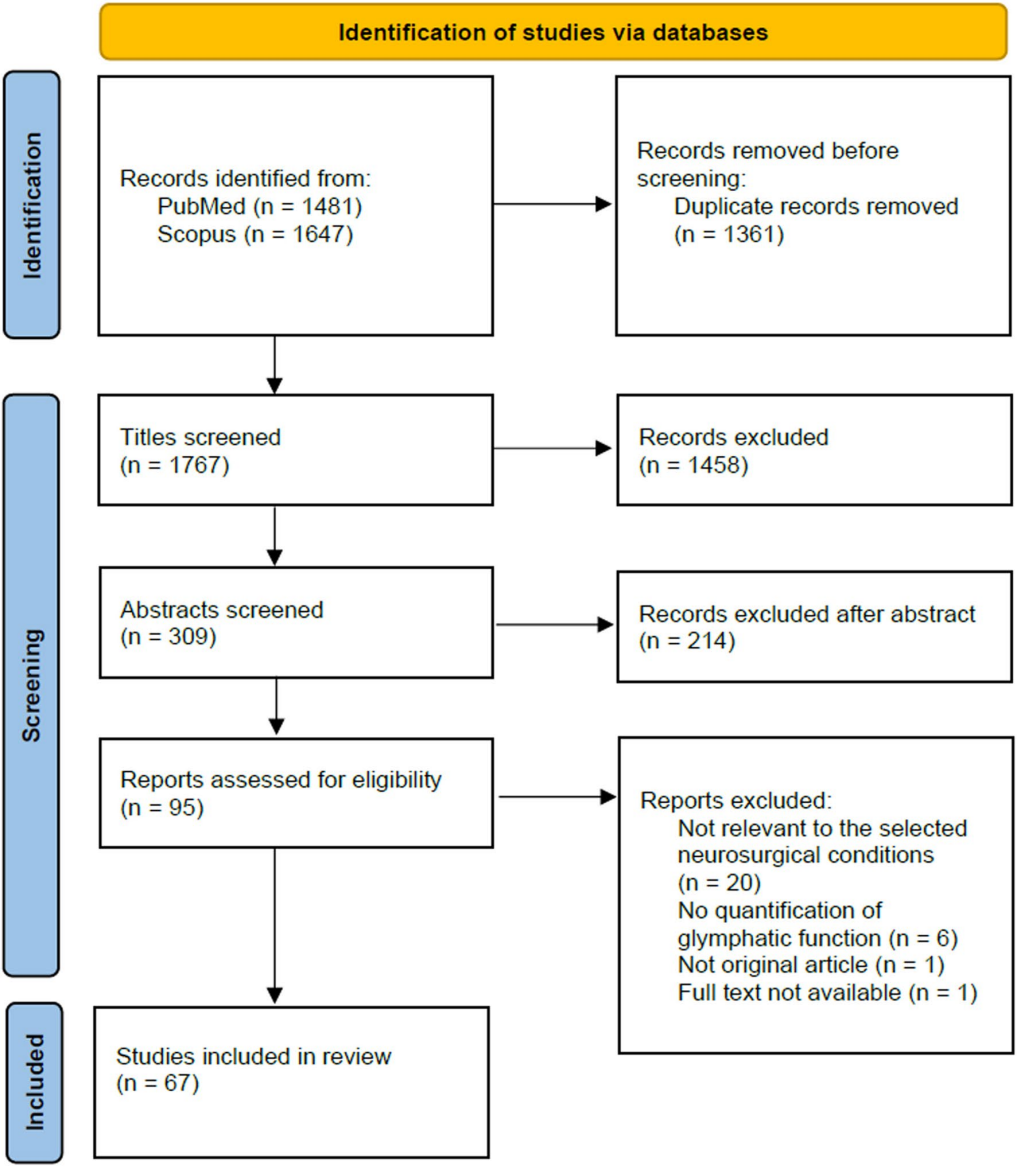
Flow chart of study inclusion

The following data were retrieved from the full text of the papers: first author and publication year, species, number of subjects, method for quantifying glymphatic function, and main result. These data were systematically tabulated and collaboratively reviewed by all team members. The process of article selection is reported in Fig. 1.

**Fig. 2:**
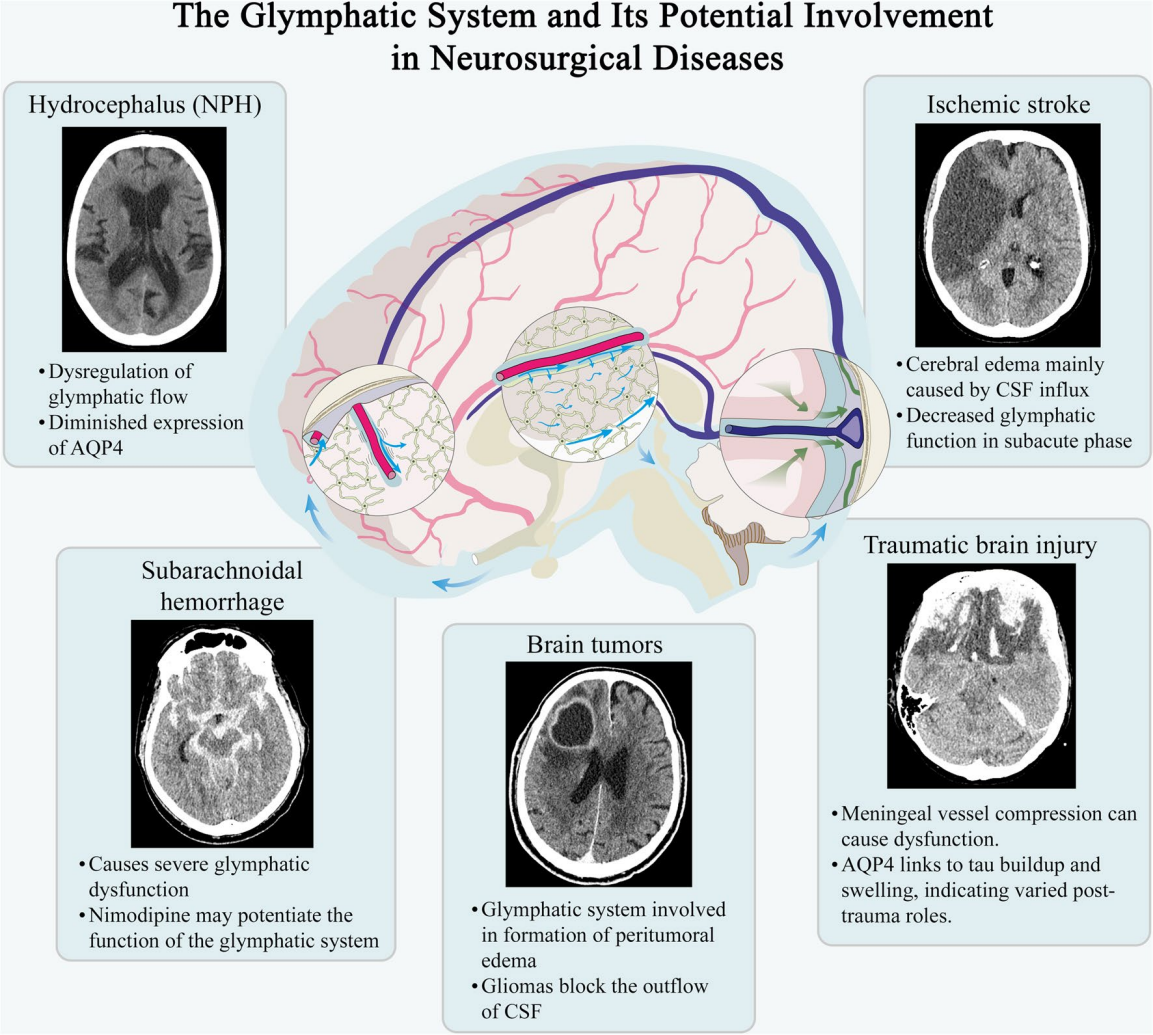
Overview of glymphatic system dysfunction in various neurosurgical conditions. This figure illustrates the involvement of the glymphatic system, which is responsible for waste clearance in the central nervous system, in several neurosurgical diseases. Each panel displays a computed tomography (CT) scan depicting a different pathological condition, accompanied by bullet points summarizing the associated changes in glymphatic function. In the center of the figure is a schematic representation of the glymphatic pathway, highlighting peri-arterial cerebrospinal fluid influx, interstitial solute movement, and solute drainage mechanisms. AQP4, aquaporin-4; CSF, cerebrospinal fluid; NPH, normal pressure hydrocephalus

## Results

### Search results

From the search conducted using the criteria outlined in the “Materials and methods” section, we identified 1767 unique articles. A total of 67 articles were included, focusing on the glymphatic system with neurosurgical relevance. These articles were subdivided into categories corresponding to neurosurgical conditions: idiopathic normal pressure hydrocephalus (iNPH), IIH, SAH, stroke, intracranial tumors, and TBI*.* For an overview of the article selection, see Fig. 1.

### Idiopathic normal pressure hydrocephalus

The glymphatic system’s alterations in patients with iNPH were explored in ten human studies, as summarized in Table 1 [8–17]. In four studies, intrathecal injection of gadobutrol followed by repeated MR imaging was employed to assess the function of the glymphatic system [10–12, 17]. In the study by Ringstad et al. CSF dynamics and glymphatic flow were severely altered in iNPH patients, showing delayed contrast enrichment of intrathecally administered gadobutrol in CSF spaces and PVS, retrograde flow in lateral ventricles, and delayed clearance of contrast agent in all CSF spaces in comparison with reference subjects [10]. Delayed glymphatic clearance has also been linked to dementia by the relation to accumulation of amyloid-β and visual pathway impairments typically seen in iNPH patients [11, 12]. Additionally, the clearance of intrathecally administered gadobutrol from the brain parenchyma has also been investigated as a useful biomarker in predicting clinical outcomes of shunt surgery, together with quantification of ventricular reflux [17].

**Table 1:** Studies investigating idiopathic normal pressure hydrocephalus and the glymphatic system

Paper	Main intervention (if applicable)	Species	Age	Number of subjects	Method for quantifying glymphatic function	Main results	Comments/other results
Hasan-Olive et al. [8]	iNPH patients	Human	iNPH: 70.8 ± 8.8; reference subjects: 44.0 ± 16.5	iNPH: *n* = 30; REF: *n* = 12	AQP-4 immunogold labeling in astrocytic endfeet in brain biopsies.	Lower AQP-4 labeling in astrocytic endfeet directed toward capillary membranes in iNPH patients compared to reference subjects.	No objective measurement of glymphatic system function was done.
Gastaldi et al. [9]	iNPH patients	Human	Median: 76 (65–86)	iNPH: *n* = 43; REF: *n* = 43; other neurodegenerative conditions: *n* = 35	No method.	AQP-4 autoantibodies were not associated with iNPH, no iNPH patients has AQP-4 autoantibodies in serum och CSF.	
Ringstad et al. [10]	iNPH patients	Human	iNPH: 71.3 ± 8.1; reference subjects: 41.1 ± 13.0	iNPH: *n* = 15; REF: *n* = 8	Intrathecal gadobutrol injection followed by repeated MRI imaging.	Slower enrichment of gadobutrol in several CSF spaces in iNPH patients in comparison with controls.	All results together indicate glymphatic dysfunction in iNPH patients.
Eide and Ringstad [11]	iNPH patients	Human	iNPH: 70.4 ± 7.5; reference subjects: 38.6 ± 14.6	iNPH: *n* = 30; REF: *n* = 8	Intrathecal gadobutrol injection followed by repeated MRI imaging.	Gadobutrol was present in a higher degree in the entorhinal cortex, CSF, and subcortical white matter in the entorhinal cortex 24 h after injection in comparison with reference subjects.	The pathophysiological alterations suggest a dysfunction in the glymphatic system and may contribute to the development of dementia in iNPH.
Jacobsen et al. [12]	iNPH patients	Human	iNPH: 71.0 ± 6.2; reference subjects: 49.6 ± 11.2	iNPH: *n* = 31; REF: *n* = 13	Intrathecal gadobutrol injection followed by repeated MRI imaging and image analysis of the visual pathway.	Delayed clearance of gadobutrol in several parts along the visual pathway in iNPH patients in comparison with controls. Delay of tracer penetration in the optic chiasm and optic tract in iNPH patients in comparison with controls.	An analysis of ICP also showed an association between pulsatile ICP-wave amplitude and a decrease in contrast agent penetration.
Eide et al. [17]	iNPH patients	Human	71.7 ± 5.8	*n* = 95	Intrathecal gadobutrol injection followed by repeated MRI imaging.	0.25 mmol was the lowest sufficient dose to maintain diagnostic capacity in the two MRI biomarkers examined in the study. Enhancing the magnetic field strength of the MRI resulted in a greater signal boost compared to increasing the gadobutrol dosage.	The two MRI biomarkers used were: (1) Clearance of gadobutrol from the CSF 24 and 48 h after intrathecal injection. (2) Determination of the grade of ventricular CSF (contrast agent) reflux.
Yokota et al. [13]	iNPH patients	Human	iNPH: 75.3 ± 7.3; pseudoNPH (piNPH): 75.7 ± 9.4; reference subjects: 75.7 ± 8.4	iNPH: *n* = 12; piNPH: *n* = 12; REF: *n* = 12	DTI-ALPS.	piNPH patients had significantly lower ALPS index than the control group. iNPH patients had significantly lower ALPS index than both the control group and the piNPH patients.	A decrease in ALPS index is highly indicative of glymphatic dysfunction.
Kikuta et al. [14]	iNPH patients	Human	iNPH: 75.22 ± 5.12	iNPH: *n* = 9	DTI-ALPS.	iNPH patients that responded positively to shunt treatment (responders) had significantly lower ALPS index postoperatively than preoperatively, and this was not seen in non-responders.	These results may indicate a recovery of the glymphatic system after shunt surgery in responders.
Eide [15]	iNPH patients	Human	iNPH: 66.2 ± 5.3, controls 55.6 ± 12.0	iNPH: *n* = 27, controls *n* = 8	Superficial cortical layers were examined using light microscope. Additionally, overnight ICP monitoring was performed.	iNPH patients brain cortex examination showed reduced expression of AQP4 expression at astrocytic endfeet and showed abnormally high pulsatile ICP when measured overnight.	iNPH is characterized by cellular changes in the glio-neurovascular interface. This is suggested by authors to possibly reflect pathophysiology of the condition.
Georgiopoulos et al. [16]	iNPH patients	Human	iNPH: 77 years, controls 73 years	iNPH: *n* = 30, healthy controls *n* = 27	DTI-ALPS.	Lower ALPS index score was found in iNPH patients compared with healthy controls. Healthy female controls had a higher ALPS index score than males.	ALPS index could serve as a marker of severity of iNPH but could be influenced by biological gender and needs further validation.

*CSF*, cerebrospinal fluid; *DTI-ALPS*, diffusion tensor imaging along the perivascular space; *iNPH*, idiopathic normal pressure hydrocephalus

The DTI-ALPS (diffusion tensor imaging-along the perivascular space) index has also been used to evaluate the brain’s glymphatic function in iNPH patients. The technique assesses the movement of interstitial fluid along the PVS with diffusion MRI. It was observed that iNPH patients exhibit a notably reduced ALPS index compared to control subjects, signifying impaired glymphatic activity [13, 16]. Furthermore, a significant increase in the ALPS index was seen in the iNPH patients responding positively to lumboperitoneal shunt surgery, something not seen in non-responders, suggesting improvement of the glymphatic function [14]. Additionally, research have shown that AQP4 expression was reduced in the astrocytic endfeet of perivascular astrocytes in iNPH patients [8, 15], and AQP4 autoantibodies were ruled out as a cause of this reduction [9].

### Idiopathic intracranial hypertension

Research on glymphatic dysfunction in IIH has been the focus of three papers, all involving human subjects [18–20]. For a complete overview, see Table 2. In the study by Eide et al. investigators used intrathecally administered gadobutrol, an MRI contrast agent, to trace CSF after being administered intrathecally [18]. Over 48 h, standardized T1 MRI scans were performed on 15 IIH patients and 15 matched reference individuals to study tracer distribution in the brain. Gadobutrol showed increased accumulation in the brain parenchyma of IIH patients and took longer to clear than in reference subjects which could indicate dysfunctional glymphatic activity among individuals with IIH.

**Table 2:** Studies investigating idiopathic intracranial hypertension and the glymphatic system

Paper	Main intervention	Species	Age	Number of subjects	Method for quantifying glymphatic function	Main results	Comments/other results
Eide et al. [18]	IIH patients	Human	IIH: 35.6 ± 12.2; REF: 32.1 ± 6.5	IIH: *n* = 15; REF: *n* = 15	Intrathecal injection of gadobutrol and MRI.	Higher enrichment and late clearance of gadobutrol in IIH patients than reference subjects.	IIH patients with pathological pulsatility of ICP were observed to have even higher enrichment and later clearance of gadobutrol in the brain parenchyma than IIH patients without pathological ICP pulsatility.
Jones et al. [19]	IIH patients	Human	IIH: 34.8 ± 9.2; REF: 37.8 ± 13.5	IIH: *n* = 32; REF: *n* = 21	Quantification of PVS visibility on MRI (PVS burden).	Higher PVS burden in patients with IIH than reference subjects in centrum semiovale and basal ganglia.	The results may indicate a role of glymphatic dysfunction in IIH, although no objective quantification of glymphatic function was carried out.
Liu et al. [20]	IIH patient	Human	39 years	*n* = 1	Normalizing of ICP and improvement clinical symptoms.	Patient with IIH, bilateral sinus transversus stenosis, and CSVD. After antihypertensive, antiplatelet, antisclerotic, and homocysteine-lowering therapies, the symptoms regressed and ICP normalized.	Authors suggest CSVD in patients with IIH and venous sinus stenosis disturbs the compensatory effect of glymphatic system in patients with IIH.

*CSVD*, cerebral small vessel disease; *IIH*, idiopathic intracranial hypertension; *PVS*, perivascular space

Furthermore, Jones et al. evaluated the number of visible PVS in 36 patients with IIH and in 19 controls, using high-resolution pre-contrast T2- and T1-weighted images [19]. An increased number of dilated PVS was found in patients with IIH compared with controls which has been suggested as a potential indicator of glymphatic system dysfunction.

### Subarachnoid hemorrhage

The impact of the glymphatic system has also been studied in relation to SAH. An overview of the 13 studies can be found in Supplemental Table 1, which includes 11 animal studies, 1 study with human participants, and 1 study with both human and animal subjects [21–33].

In mice, decreased fluorescent tracer penetration and enlarged PVS, along with an increase in P-tau (phosphorylated tau) and tau-protein presence, decreased neurological scoring, and AQP4 depolarization, were observed after SAH, indicating glymphatic dysfunction [21, 23]. Deletion of the AQP4 gene in mice and rats has also been observed to aggravate glymphatic dysfunction, neuronal damage, and neurological deficits following SAH [24, 33]. Furthermore, induced overexpression of AQP4 using a viral vector resulted in improved depolarization of AQP4 and subsequent improved neurobehavioral ability in mice [27]. SAH has also been observed to cause severe glymphatic dysfunction in the acute phase [25, 26, 28]. In mice, studies point toward a decreased glymphatic function due to brain tissue factor, fibrin deposits, and fibrinogen filling the PVS [26]. Furthermore, intraventricular injection of tissue factor antibodies caused an increase in CSF flow following SAH [26].

The presence of fibrin in the PVS along with a decrease in parenchymal penetration of contrast agent has also been observed in non-human primates [29]. In humans, perivascular presence of blood clots has been observed after aneurysmal SAH as well as enlarged PVS in centrum semiovale and the basal ganglia, indicating glymphatic dysfunction [30]. Interestingly, Luo et al. found that tissue plasminogen activator (tPA) improved neurological outcomes in mice of SAH after 7 days due to an improvement of the glymphatic clearance [28].

Nimodipine, a calcium channel antagonist commonly used as a prophylactic agent against cerebral vasospasm in SAH, was investigated in relation to the glymphatic system in mice [31]. The results indicated that glymphatic function improved after nimodipine administration and eliminated the effects of edema and neurological dysfunction after SAH. The authors therefore suggest that nimodipine has a neuroprotective role after SAH.

Additionally, treatment with pituitary adenylate cyclase-activating polypeptide has been shown to increase glymphatic function after SAH and alleviate the subsequent increase in intracranial pressure and brain water content following SAH [32].

### Stroke

Ischemic and hemorrhagic strokes have also been investigated in terms of their effect on the glymphatic system [25, 34–46]. See Supplemental Table 2 for an overview of the 14 included articles spanning 10 animal and 4 human studies.

In mice, middle carotid artery occlusion (MCAO) resulted in reduced parenchymal penetration of both fluorescent tracer and MRI contrast agent, along with an impairment of perivascular polarity of AQP4 and impairment of glymphatic system in thalamus [42, 45]. The post-stroke edema was also investigated in mice, where intravenous and intracisternal administration of ^22^Na and ^3^H-mannitol revealed a predominant influx of CSF into the edematous tissue from the lateral ventricles, cisterna magna, and the PVS [35]. In the same study, vasoconstriction following MCAO resulted in an enlargement of PVS, causing an increased influx of CSF and contrast agent contributing to the edema.

Glymphatic function was investigated using TGN-020 to mediate AQP4 inhibition and was correlated with a reduction in brain edema post-MCAO in rats and promoting neurological recovery at 14 days post-stroke [36]. After MCAO in rats, no difference in contrast agent penetration of the brain was seen in the acute phase, although significantly impaired parenchymal penetration and clearance was seen in the subacute phase [37]. In humans, ischemic stroke resulted in a lower ALPS index in the lesion hemisphere compared to both the contralateral hemisphere in the same patient and the ipsilateral hemisphere in reference subjects [38]. Furthermore, ALPS values were less affected in smaller strokes, and with longer follow-up post-stroke [38].

In patients with spontaneous ICHs, a higher ALPS index was observed. Furthermore, an increased visibility of PVS, known as PVS burden, was noted on MRI in the centrum semiovale of these patients, regardless of the presence of cerebral amyloid angiopathy [39]. Additionally, a greater number of PVS correlated with more amyloid deposits in the brain, indicating a link between cerebral amyloid angiopathy and glymphatic dysfunction after spontaneous ICH [39]. Ligation and removal of the cervical lymph nodes (cervical lymphatic blockage) in rats with induced ICH resulted in higher brain water content and lower levels of AQP4 expression than only induction of ICH without cervical lymphatic blockage [40]. In a case study, an idiopathic subdural hematoma in a pig resulted in an impairment of CSF tracer distribution in the whole brain [41].

### Intracranial tumors

The link between brain tumors and the glymphatic system has been explored in six studies, as detailed in Table 3 [47–52]. Of these, three studies were conducted on human subjects and three involved animal models.

**Table 3:** Studies investigating intracranial tumors and the glymphatic system

Paper (author and year)	Type of tumor	Species	Age	Number of subjects	Method for quantifying glymphatic function	Main results	Comments/other results
Toh and Siow [47]	Glioma	Human	47.4 ± 15.5	*n* = 201	Calculation of ALPS index using DTI-ALPS.	Grade 4 gliomas were associated with a lower ALPS index than grade II and grade III gliomas. No significant difference in the ALPS index value was observed between grade II and grade III gliomas.	The presence of an IDH mutation was associated with higher ALPS index values. Volume of peritumoral edema was negatively correlated with the ALPS index value.
Ma et al. [48]	Induced glioma via injection of GL261 cells into the right striatum	Mouse	2–3 months	N/A	Intracisternal tracer injection followed by analysis of signal intensity in the saphenous vein, dcLNs and mandibular lymph nodes, and fluorescence imaging of cranial nerves after 60 min. Intracisternal Gadospin D injection followed by MRI imaging.	Significantly reduced signal intensity in saphenous vein, dcLNs, and mandibular lymph nodes in mice with glioma in comparison with control mice. Decreased CSF flow along several cranial nerves in mice with glioma. Complete blockage of clearance of intracisternal Gadospin D in mice with gliomas.	An increase in spinal outflow of CSF was shown in mice with gliomas, indicating a redirection of CSF outflow pathways caused by the gliomas. All results together indicate glymphatic and cerebral lymphatic dysfunction following glioma induction.
Xu et al. [49]	Induced glioma via injection of rat glioma C6 cells into the right striatum	Rat	6 weeks	N/A	Intracisternal gadobutrol injection followed by repeated MRI imaging. Intracisternal Evans blue injection followed by macroscopic analysis.	Increased MRI signal in the olfactory bulbs 3–4 h after gadobutrol injection. Retrograde tracer flow into the ventricles in rats. Lower tracer and gadobutrol influx into the glioma than in healthy tissue, and mostly limited to the periphery of the tumor. Evans blue and gadobutrol signal strength was also higher in the side contralateral to the tumor in comparison to the tumor side.	AQP-4 expression in and around the glioma was also decreased. Results indicate that glymphatic flow is obstructed and redirected in rats with gliomas, and healthy tissue seems to compensate for the glioma-containing side in terms of glymphatic outflow pathways.
Toh et al. [50]	Brain metastases	Human	56.9 ± 11.6	*n* = 56	Calculation of ALPS index using DTI-ALPS.	Peritumoral brain edema (PTBE) was negatively correlated with ALPS index.	Negative correlation with ALPS index values could be indicative of a connection between PTBE and glymphatic dysfunction.
Toh et al. [51]	Meningioma	Human	58.8 ± 13.5	*n* = 80	Calculation of ALPS index using DTI-ALPS.	Meningiomas without PTBE were associated with a higher ALPS than normal patients and meningiomas with PTBE.	Decrease in glymphatic function may be linked to the formation of PTBE.
Li et al. [52]	Rats with spontaneous pituitary tumor	Rat	22–24 months	*n* = 14	Intracisternal contrast injection, evaluated with MRI T2-weighted and dynamic 3D T1-weighted.	A lower incidence of enhancement of glymphatic influx nodes and pituitary and pineal recesses in rats with pituitary tumors.	The glymphatic system seems to be affected in rats with spontaneous pituitary tumor.

*dcLNs*, draining cervical lymph nodes; *DTI-ALPS*, diffusion tensor imaging along the perivascular space; *PTBE*, peritumoral brain edema

Gliomas have been associated with lower ALPS indexes than reference subjects in humans, where grade 4 *IDH*-wild-type glioblastomas had the lowest ALPS indexes [47]. Furthermore, *IDH*-mutated gliomas showed a higher ALPS index compared to *IDH*-wild-type gliomas. Induced gliomas in mice have also been observed to block perineural and lymphatic outflow of CSF, shown through lower concentrations of intracisternally injected fluorescent tracer in the saphenous vein, deep cervical lymph nodes and mandibular lymph nodes, and a lower perineural MRI signal [48]. Xu et al. showed that the glymphatic flow was hindered and redirected in rats with gliomas, along with a decreased expression of AQP4 in the astrocytes [49]. Low ALPS index has also been correlated with peritumoral brain edema (PTBE) in patients with brain metastases and meningiomas. This provides evidence for the role of glymphatic dysfunction in the formation of PTBE [50, 51]. The glymphatic system also seems to be affected in rat model with spontaneous pituitary tumor, which could result in cognitive impairment [52].

### Traumatic brain injury

There has been a significant interest to study glymphatic function in TBI. For a comprehensive overview, refer to Supplementary Table 3 [53–74]. Among the 22 studies included, 12 primarily utilized animal models to explore the effects of TBI on the glymphatic system.

Repetitive mild TBI (mTBI) was shown to result in increased penetration of intracisternally injected contrast agent in amygdala, hypothalamus, hippocampus, and olfactory bulb. This was coupled with a decreased efflux rate, suggesting impaired glymphatic clearance compared to control mice [53]. Similar results were shown in the study by Li et al. where moderate TBI resulted in a delay of both penetration and clearance of intracisternally injected gadobutrol in several brain areas, but most pronounced in the olfactory bulb [54].

In another mouse study, a decrease in penetration of fluorescent tracer was observed following TBI, combined with decreased AQP4 perivascular polarization, increased glial scarring, and increased P-tau accumulation [55]. Additionally, knockout of AQP4 resulted in further increase of P-tau accumulation. Lui et al. found that AQP4 knockout in mice showed improved outcome regarding cognitive functions in mice with TBI in comparison to wild-type mice with TBI, including less brain edema, higher BBB integrity, higher neurological and cognitive performance, decreased amyloid beta levels, and a decrease in inflammatory cytokines [56]. The authors speculate that the AQP4 deficiency protected the blood-brain barrier integrity and clearance of amyloid-β. Knockout of angiotensin II type receptor was found to have a positive effect on glymphatic function in mice with TBI, resulting in less perivascular depolarization of AQP4, decreased infarction volume and brain water content, and higher clearance of amyloid-β than wild-type mice [57]. Furthermore, in the study by Bolte et al. meningeal lymphatics are also negatively affected by TBI, shown by long-term changes in morphology and function following mTBI in mice [58].

The effects of altering the glymphatic system on the detection of common TBI biomarkers were also investigated in mice. Approaches such as AQP4 knockout, cisternostomy, treatment with acetazolamide, and sleep deprivation all resulted in a reduction in clearance of injected tracer substance and blocked the glymphatic clearance [59]. Even though the mechanisms resulted in inhibition of clearance of radiotracer, the blood serum biomarkers (S100beta, GFAP, and NSE) also decreased in a similar fashion.

As for the human studies, the correlation between TBI, sleep, and glymphatic function has been examined. Opel et al. observed a correlation between the visibility of PVS in MRI and sleep disturbance in patients with TBI [60]. In another study, Piantino et al. showed similar results in military veterans, where a positive correlation between the amount of mTBIs and PVS burden was shown. The authors also found an interaction between mTBI and PVS burden and suggest the increased PVS burden may be a sign of glymphatic clearance dysfunction which also seems to be associated with the severity of post-concussion symptoms such as balance problems, dizziness, and nausea [61].

One study tested a hypothesis of “CSF shift edema,” whereby a positive pressure gradient between the basal cisterns and the brain parenchyma is created by a head injury, pushing CSF through the PVS into the brain parenchyma, causing edema. A basal cisternostomy would reverse this pressure gradient, potentially alleviating brain edema. When combining decompressive hemicraniectomy with either partial or complete basal cisternostomy, the authors found that a low intracisternal pressure was associated with survival, whereas an increase in intracisternal pressure was associated with clinical worsening and death. However, the sample size was too small for any meaningful statistical analysis [63].

The study by Chen et al. describes a surgical technique aimed at improving outcomes for patients with chronic subdural hematomas (CSDH). This technique involves fenestration of the inner membrane after the removal of CSDH and it is hypothesized that this technique may enhance glymphatic flow postoperatively [74]. In mice with TBI, both exogenous IL-33 and the activation of cerebral GLP-1 receptors (GLP-1R) have been shown to have therapeutic effects by enhancing the function of the glymphatic system. IL-33 improves motor and cognitive abilities by promoting lymphatic drainage and clearing toxic metabolites. Similarly, activating GLP-1R helps to restore glymphatic transport and reduce cognitive impairment [68, 69].

## Discussion

In this scoping review, we present the current body of literature regarding the glymphatic system’s involvement in several neurosurgical conditions, highlighting its crucial role in the underlying pathophysiology of these disorders and its emerging potential as a therapeutic target. Our synthesis reveals that the glymphatic system is involved in the regulation of brain swelling and CSF dynamics across a spectrum of neurosurgical pathologies and the AQP4 channels emerge as central regulators in this intricate system. Advanced imaging modalities, including DTI and MRI with intrathecally contrast agents, have been instrumental in elucidating the alterations in glymphatic function, extending these observations to human subjects. These studies collectively suggest that dysfunction within the glymphatic system may not only contribute to the progression of neurosurgical diseases but may also, through its restoration, offer a conduit for therapeutic intervention. The interest in clinically exploring the glymphatic system is growing, as evidenced both by the results of the current review and by planned and ongoing studies listed on clinicaltrials.gov. These include research on glymphatic function in patients undergoing ventriculoperitoneal shunt surgery, investigations into the glymphatic pathway using brain imaging, and studies focusing on the glymphatic system in epilepsy (clinicaltrials.gov visited 2024-01-02).

In iNPH, studies that use MR imaging with intrathecal contrast have shown significant dysregulation of glymphatic flow, evidenced by delayed clearance of the contrast agent and abnormal CSF dynamics. Furthermore, diminished expression of AQP4 in the perivascular astrocytic endfeet may provide a pathophysiological explanation for the glymphatic dysfunction seen in iNPH. Selecting iNPH patients who are likely to respond well to shunt treatment remains challenging; however, the clearance of intrathecal gadobutrol could potentially serve as a biomarker to improve patient selection. Moreover, the ALPS index may offer a means to evaluate glymphatic function and could be useful in a clinical setting, particularly in cases where the response to shunt treatment is uncertain.

Similarly, in IIH, the glymphatic system’s impairment is suggested by the prolonged presence of intrathecal gadobutrol and increased visibility of PVS on MRI, which could reflect a failure in efficient waste clearance. These findings may have therapeutic implications, particularly in the optimization of current treatment strategies and the development of novel interventions.

In the context of SAH, our review has identified evidence of glymphatic system dysfunction, due to accumulations of fibrin and fibrinogen in the PVS. The therapeutic benefits of enhancing glymphatic function are highlighted by the positive outcomes associated with nimodipine and tPA treatments. This underscores the importance of preserving glymphatic integrity after the ictus. The potential effects of tranexamic acid (TXA) on glymphatic function post-SAH are not yet understood. It is interesting to speculate how the administration TXA, to reduce the risk of rebleeding, affects the function of the glymphatic system after SAH. To date, there is a lack of research exploring whether TXA’s antifibrinolytic properties could adversely affect glymphatic function. In this light, the recent findings by Wolf et al. are particularly intriguing, as they suggest that prophylactic lumbar drainage after aneurysmal SAH may improve patient outcomes by facilitating CSF clearance and reducing the likelihood of secondary infarctions and unfavorable 6-month prognoses [75].

The role of the glymphatic system in stroke recovery is similarly noteworthy. Studies focusing on MCAO revealed several key findings: enlargement of PVS [35], reduced contrast agent clearance during the subacute phase [37], and an association between increased PVS burden and amyloid deposition. All these findings may have a common explanation through impaired glymphatic function [39]. The therapeutic modulation of this system, through agents such as the AQP4 inhibitor TGN-020, suggests a promising avenue for enhancing neurological recovery and reducing cognitive deficits. The potential benefits of CSF drainage in the aftermath of a stroke present an intriguing area for exploration. Considering that cerebral edema may be predominantly composed of CSF, the implementation of drainage systems could feasibly mitigate the progression of edema. This approach warrants further investigation as it may offer a therapeutic avenue to alleviate post-stroke complications.

Concerning the relationship between brain tumors and the glymphatic system, it is noteworthy to mention that the impairment of glymphatic functions correlates with the increasing malignancy of brain tumors. This dysfunction is particularly pronounced in diffuse gliomas, where it is associated with IDH mutation status and the WHO grade of the tumor. It is plausible to speculate that the differential response of PTBE to corticosteroids, as opposed to edema from other causes such as stroke or TBI, could be due to variations in the involvement of the glymphatic system. This hypothesis suggests that an improved understanding of glymphatic function in the context of brain tumors could potentially inform more targeted therapeutic strategies and contribute to better management of tumor-associated edema.

TBI and its connection to the glymphatic system have been extensively studied, and research indicates that glymphatic dysfunction after TBI can contribute to long-term cognitive impairment. This dysfunction may partly stem from the compression of meningeal lymphatic vessels that occurs during head trauma, leading to an accumulation of harmful byproducts. Animal studies support this, showing decreased efflux and increased accumulation of imaging contrasts in brain regions critical for cognitive function, hinting at a compromised glymphatic flow.

AQP4 channels have emerged as a significant factor also in TBI pathology. While some studies suggest that a lack of AQP4 polarization leads to an increase in tau-protein accumulation, indicative of brain damage, others point to less edema and better neurological function in the absence of AQP4. These seemingly contradictory findings may reflect the multifaceted roles AQP4 plays at different stages post-injury. Angiotensin II receptor knockouts have led to improved outcomes, suggesting that modulation of this pathway could have therapeutic potential. Similarly, studies have demonstrated that interventions by the administration of IL-33 or GLP-1R agonists can enhance waste clearance, reduce edema, and improve cognitive outcomes. These interventions highlight the glymphatic system’s therapeutic potential in TBI management.

Moreover, the glymphatic system’s connection to sleep has implications for TBI recovery, with increased PVS visibility on MRI scans potentially signaling glymphatic impairment and correlating with the severity of post-concussion symptoms. It raises intriguing questions about the effects of frequently waking patients for monitoring after a head injury on glymphatic function, especially given its enhanced activity during sleep. Future research should investigate how such medical practices may affect glymphatic clearance and patient recovery.

### Strengths/limitations

This review systematically consolidates a wide range of studies to provide a comprehensive understanding of the glymphatic system’s role in various neurosurgical conditions, representing a significant strength of this work. Our methodological approach and inclusion of both animal and human studies allow for a multifaceted perspective on the glymphatic system’s functions and its potential therapeutic targets. However, there are limitations to consider.

There remain significant uncertainties in our understanding of this system. For instance, the drainage pathway from the perivenous spaces to the meningeal lymphatics is still not well described. Additionally, the mechanisms driving solutes and waste from interstitial tissue along perivascular pathways are not fully understood. Furthermore, while AQP4 has been implicated in perivascular fluid transport, the precise nature of its involvement remains elusive. These areas of uncertainty underscore the need for continued research to fully elucidate the complexities of the glymphatic system.

The studies included are heterogeneous in terms of design, measurement techniques, and outcomes, which may affect the generalizability of the findings. It is also important to consider that there are uncertainties associated with methodologies employed to quantify glymphatic system function in humans. The DTI-ALPS technique, used in many of the papers, assesses the anisotropy of water diffusion along PVS in white matter. However, the glymphatic system mainly involves the movement of substances along PVS and in the interstitium in gray matter. Consequently, the precise correlation between DTI-ALPS measurements and glymphatic functionality remains intricate and not fully understood. Furthermore, while the majority of the data derive from animal studies, providing invaluable insights, the extrapolation of these findings to human pathology necessitates caution, as direct applicability may be limited.

## Conclusion

In summarizing the current literature, this scoping review elucidates the integral role of the glymphatic system within various neurosurgical conditions, highlighting its influence on brain edema and CSF dynamics. The compilation of findings emphasizes the system’s widespread impact and the modulatory role of AQP4 channels as a common factor across different neurosurgical conditions. Despite the advancements in imaging and biomarker identification, the translation of these findings into clinical practice necessitates further human studies. The glymphatic system has the potential to lead to new treatments, especially by targeting AQP4 channels. As our understanding of this complex system grows, it offers the potential for developing new treatment strategies that could improve patient care and outcomes in neurosurgery.

## Supplementary information

ESM 1

ESM 2

ESM 3

## Data Availability

Not applicable.

## References

[1] Weed LH (1914) Studies on cerebro-spinal fluid. No. III: the pathways of escape from the Subarachnoid Spaces with particular reference to the Arachnoid Villi. J Med Res 31(1):51–91 19972194 PMC2094443

[2] Iliff JJ, Wang M, Liao Y et al (2012) A paravascular pathway facilitates CSF flow through the brain parenchyma and the clearance of interstitial solutes, including amyloid β. Sci Transl Med 4(147):147ra111. 10.1126/scitranslmed.3003748 22896675 10.1126/scitranslmed.3003748 PMC3551275

[3] Silva I, Silva J, Ferreira R, Trigo D (2021) Glymphatic system, AQP4, and their implications in Alzheimer’s disease. Neurol Res Pract 3(1):5. 10.1186/s42466-021-00102-7 33499944 10.1186/s42466-021-00102-7 PMC7816372

[4] Eide PK, Vatnehol SAS, Emblem KE, Ringstad G (2018) Magnetic resonance imaging provides evidence of glymphatic drainage from human brain to cervical lymph nodes. Sci Rep 8(1):7194. 10.1038/s41598-018-25666-4 29740121 10.1038/s41598-018-25666-4 PMC5940793

[5] Møllgård K, Beinlich FRM, Kusk P et al (2023) A mesothelium divides the subarachnoid space into functional compartments. Science 379(6627):84–88. 10.1126/science.adc8810 36603070 10.1126/science.adc8810

[6] Reddy OC, van der Werf YD (2020) The sleeping brain: harnessing the power of the glymphatic system through lifestyle choices. Brain Sci 10(11):868. 10.3390/brainsci10110868 33212927 10.3390/brainsci10110868 PMC7698404

[7] Bohr T, Hjorth PG, Holst SC et al (2022) The glymphatic system: current understanding and modeling. iScience 25(9):104987. 10.1016/j.isci.2022.104987 36093063 10.1016/j.isci.2022.104987 PMC9460186

[8] Hasan-Olive MM, Enger R, Hansson HA et al (2019) Loss of perivascular aquaporin-4 in idiopathic normal pressure hydrocephalus. Glia 67(1):91–100. 10.1002/glia.23528 30306658 10.1002/glia.23528

[9] Gastaldi M, Todisco M, Carlin G et al (2020) AQP4 autoantibodies in patients with idiopathic normal pressure hydrocephalus. J Neuroimmunol 349:577407. 10.1016/j.jneuroim.2020.577407 33032017 10.1016/j.jneuroim.2020.577407

[10] Ringstad G, Vatnehol SAS, Eide PK (2017) Glymphatic MRI in idiopathic normal pressure hydrocephalus. Brain: A. J Neurol 140(10):2691–2705. 10.1093/brain/awx191 10.1093/brain/awx191 PMC5841149 28969373

[11] Eide PK, Ringstad G (2019) Delayed clearance of cerebrospinal fluid tracer from entorhinal cortex in idiopathic normal pressure hydrocephalus: a glymphatic magnetic resonance imaging study. J Cereb Blood Flow Metab 39(7):1355–1368. 10.1177/0271678x18760974 29485341 10.1177/0271678X18760974 PMC6668515

[12] Jacobsen HH, Sandell T, Jørstad ØK, Moe MC, Ringstad G, Eide PK (2020) In vivo evidence for impaired glymphatic function in the visual pathway of patients with normal pressure hydrocephalus. Invest Ophthalmol Vis Sci 61(13):24. 10.1167/iovs.61.13.24 33201186 10.1167/iovs.61.13.24 PMC7683855

[13] Yokota H, Vijayasarathi A, Cekic M et al (2019) Diagnostic performance of glymphatic system evaluation using diffusion tensor imaging in idiopathic normal pressure hydrocephalus and mimickers. Curr Gerontol Geriatr Res 2019:5675014. 10.1155/2019/5675014 31320896 10.1155/2019/5675014 PMC6609364

[14] Kikuta J, Kamagata K, Taoka T et al (2022) Water diffusivity changes along the perivascular space after lumboperitoneal shunt surgery in idiopathic normal pressure hydrocephalus. Front Neurol 13:843883. 10.3389/fneur.2022.843883 35295837 10.3389/fneur.2022.843883 PMC8918529

[15] Eide PK (2022) Cellular changes at the glia-neuro-vascular interface in definite idiopathic normal pressure hydrocephalus. Front Cell Neurosci 16:981399. 10.3389/fncel.2022.981399 36119130 10.3389/fncel.2022.981399 PMC9478415

[16] Georgiopoulos C, Tisell A, Holmgren RT et al (2023) Noninvasive assessment of glymphatic dysfunction in idiopathic normal pressure hydrocephalus with diffusion tensor imaging. J Neurosurg 8:1–9. 10.3171/2023.6.Jns23260 10.3171/2023.6.JNS23260 37724800

[17] Eide PK, Lashkarivand A, Hagen-Kersten ÅA et al (2022) Intrathecal contrast-enhanced magnetic resonance imaging of cerebrospinal fluid dynamics and glymphatic enhancement in idiopathic normal pressure hydrocephalus. Front Neurol 13:857328. 10.3389/fneur.2022.857328 35463139 10.3389/fneur.2022.857328 PMC9019061

[18] Eide PK, Pripp AH, Ringstad G, Valnes LM (2021) Impaired glymphatic function in idiopathic intracranial hypertension. Brain Comm 3(2):fcab043. 10.1093/braincomms/fcab043 10.1093/braincomms/fcab043 PMC8253298 34235434

[19] Jones O, Cutsforth-Gregory J, Chen J, Bhatti MT, Huston J, Brinjikji W (2021) Idiopathic intracranial hypertension is associated with a higher burden of visible cerebral perivascular spaces: the glymphatic connection. AJNR Am J Neuroradiol 42(12):2160–2164. 10.3174/ajnr.A7326 34824096 10.3174/ajnr.A7326 PMC8805760

[20] Liu W, Jia L, Xu L et al (2023) Idiopathic intracranial hypertension in patients with cerebral small vessel disease: a case report. Medicine 102(1):e32639. 10.1097/md.0000000000032639 36607854 10.1097/MD.0000000000032639 PMC9829262

[21] Hou C, Li J, Wang B et al (2022) Dynamic evolution of the glymphatic system at the early stages of subarachnoid hemorrhage. Front Neurol 13:924080. 10.3389/fneur.2022.924080 35847203 10.3389/fneur.2022.924080 PMC9283644

[22] Liu E, Peng X, Ma H et al (2021) The involvement of aquaporin-4 in the interstitial fluid drainage impairment following subarachnoid hemorrhage. Front Aging Neurosci 12:611494. 10.3389/fnagi.2020.611494 33574749 10.3389/fnagi.2020.611494 PMC7870692

[23] Pu T, Zou W, Feng W et al (2019) Persistent malfunction of glymphatic and meningeal lymphatic drainage in a mouse model of subarachnoid hemorrhage. Exp Neurobiol 28(1):104–118. 10.5607/en.2019.28.1.104 30853828 10.5607/en.2019.28.1.104 PMC6401547

[24] Liu E, Sun L, Zhang Y, Wang A, Yan J (2020) Aquaporin4 knockout aggravates early brain injury following subarachnoid hemorrhage through impairment of the glymphatic system in rat brain. Acta Neurochir Suppl 127:59–64. 10.1007/978-3-030-04615-6_10 31407064 10.1007/978-3-030-04615-6_10

[25] Gaberel T, Gakuba C, Goulay R et al (2014) Impaired glymphatic perfusion after strokes revealed by contrast-enhanced MRI: a new target for fibrinolysis? Stroke 45(10):3092–3096. 10.1161/strokeaha.114.006617 25190438 10.1161/STROKEAHA.114.006617

[26] Golanov EV, Bovshik EI, Wong KK et al (2018) Subarachnoid hemorrhage-induced block of cerebrospinal fluid flow: role of brain coagulation factor III (tissue factor). J Cereb Blood Flow Metab 38(5):793–808. 10.1177/0271678x17701157 28350198 10.1177/0271678X17701157 PMC5987942

[27] Liu Y, Wang Z, Cao C et al (2022) Aquaporin 4 depolarization-enhanced transferrin infiltration leads to neuronal ferroptosis after subarachnoid hemorrhage in mice. Oxidative Med Cell Longev 2022:8808677. 10.1155/2022/8808677 10.1155/2022/8808677 PMC9233479 35761873

[28] Luo C, Yao X, Li J et al (2016) Paravascular pathways contribute to vasculitis and neuroinflammation after subarachnoid hemorrhage independently of glymphatic control. Cell Death Dis 7(3):e2160. 10.1038/cddis.2016.63 27031957 10.1038/cddis.2016.63 PMC4823962

[29] Goulay R, Flament J, Gauberti M et al (2017) Subarachnoid hemorrhage severely impairs brain parenchymal cerebrospinal fluid circulation in nonhuman primate. Stroke 48(8):2301–2305. 10.1161/strokeaha.117.017014 28526764 10.1161/STROKEAHA.117.017014

[30] Kim J, Joo B, Kim JW et al (2022) Aggravation of enlarged perivascular spaces in the centrum semiovale of patients with aneurysmal subarachnoid hemorrhage. Clin Neuroradiol 32(1):79–87. 10.1007/s00062-021-01098-y 34618170 10.1007/s00062-021-01098-y

[31] Hou C, Liu Q, Zhang H et al (2022) Nimodipine attenuates early brain injury by protecting the glymphatic system after subarachnoid hemorrhage in mice. Neurochem Res 47(3):701–712. 10.1007/s11064-021-03478-9 34792752 10.1007/s11064-021-03478-9

[32] Fang Y, Shi H, Ren R et al (2020) Pituitary adenylate cyclase-activating polypeptide attenuates brain edema by protecting blood-brain barrier and glymphatic system after subarachnoid hemorrhage in rats. Neurotherapeutics 17(4):1954–1972. 10.1007/s13311-020-00925-3 32918234 10.1007/s13311-020-00925-3 PMC7851266

[33] Liu E, Peng X, Ma H et al (2020) The involvement of aquaporin-4 in the interstitial fluid drainage impairment following subarachnoid hemorrhage. Front Aging Neurosci 12:611494. 10.3389/fnagi.2020.611494 33574749 10.3389/fnagi.2020.611494 PMC7870692

[34] Zhang C, Sha J, Cai L et al (2022) Evaluation of the glymphatic system using the DTI-ALPS index in patients with spontaneous intracerebral haemorrhage. Oxidative Med Cell Longev 2022:2694316. 10.1155/2022/2694316 10.1155/2022/2694316 PMC9277160 35847591

[35] Mestre H, Du T, Sweeney AM et al (2020) Cerebrospinal fluid influx drives acute ischemic tissue swelling. Science 367:6483. 10.1126/science.aax7171 10.1126/science.aax7171 PMC7375109 32001524

[36] Sun C, Lin L, Yin L et al (2022) Acutely inhibiting AQP4 with TGN-020 improves functional outcome by attenuating edema and peri-infarct astrogliosis after cerebral ischemia. Front Immunol 13:870029. 10.3389/fimmu.2022.870029 35592320 10.3389/fimmu.2022.870029 PMC9110854

[37] Lin L, Hao X, Li C et al (2020) Impaired glymphatic system in secondary degeneration areas after ischemic stroke in rats. J Stroke Cerebrovasc Dis 29(7):104828. 10.1016/j.jstrokecerebrovasdis.2020.104828 32404284 10.1016/j.jstrokecerebrovasdis.2020.104828

[38] Toh CH, Siow TY (2021) Glymphatic dysfunction in patients with ischemic stroke. Front Aging Neurosci 13:756249. 10.3389/fnagi.2021.756249 34819849 10.3389/fnagi.2021.756249 PMC8606520

[39] Tsai HH, Pasi M, Tsai LK et al (2021) Centrum semiovale perivascular space and amyloid deposition in spontaneous intracerebral hemorrhage. Stroke 52(7):2356–2362. 10.1161/strokeaha.120.032139 33874751 10.1161/STROKEAHA.120.032139 PMC8989045

[40] Liu X, Wu G, Tang N et al (2021) Glymphatic drainage blocking aggravates brain edema, neuroinflammation via modulating TNF-α, IL-10, and AQP4 after intracerebral hemorrhage in rats. Front Cell Neurosci 15:784154. 10.3389/fncel.2021.784154 34975411 10.3389/fncel.2021.784154 PMC8718698

[41] Shanbhag NC, Bèchet NB, Kritsilis M, Lundgaard I (2021) Impaired cerebrospinal fluid transport due to idiopathic subdural hematoma in pig: an unusual case. BMC Vet Res 17(1):250. 10.1186/s12917-021-02954-2 34284779 10.1186/s12917-021-02954-2 PMC8290550

[42] Zhang J, Zhao H, Xue Y et al (2022) Impaired glymphatic transport kinetics following induced acute ischemic brain edema in a mouse pMCAO model. Front Neurol 13:860255. 10.3389/fneur.2022.860255 35370910 10.3389/fneur.2022.860255 PMC8970176

[43] Yi T, Gao P, Hou M et al (2022) The mechanisms underlying the actions of Xuefu Zhuyu decoction pretreatment against neurological deficits after ischemic stroke in mice: the mediation of glymphatic function by aquaporin-4 and its anchoring proteins. Front Pharmacol 13:1053253. 10.3389/fphar.2022.1053253 36582539 10.3389/fphar.2022.1053253 PMC9792381

[44] Qin Y, Li X, Qiao Y et al (2023) DTI-ALPS: an MR biomarker for motor dysfunction in patients with subacute ischemic stroke. Front Neurosci 17:1132393. 10.3389/fnins.2023.1132393 37065921 10.3389/fnins.2023.1132393 PMC10102345

[45] Li C, Lin L, Sun C et al (2022) Glymphatic system in the thalamus, secondary degeneration area was severely impaired at 2nd week after transient occlusion of the middle cerebral artery in rats. Front Neurosci 16:997743. 10.3389/fnins.2022.997743 36278004 10.3389/fnins.2022.997743 PMC9582259

[46] Liu Y, Liu X, Sun P et al (2023) rTMS treatment for abrogating intracerebral hemorrhage-induced brain parenchymal metabolite clearance dysfunction in male mice by regulating intracranial lymphatic drainage. Brain Behav 13(7):e3062. 10.1002/brb3.3062 37161559 10.1002/brb3.3062 PMC10338767

[47] Toh CH, Siow TY (2021) Factors associated with dysfunction of glymphatic system in patients with glioma. Front Oncol 11:744318. 10.3389/fonc.2021.744318 34631582 10.3389/fonc.2021.744318 PMC8496738

[48] Ma Q, Schlegel F, Bachmann SB et al (2019) Lymphatic outflow of cerebrospinal fluid is reduced in glioma. Sci Rep 9(1):14815. 10.1038/s41598-019-51373-9 31616011 10.1038/s41598-019-51373-9 PMC6794292

[49] Xu D, Zhou J, Mei H et al (2021) Impediment of cerebrospinal fluid drainage through glymphatic system in glioma. Front Oncol 11:790821. 10.3389/fonc.2021.790821 35083148 10.3389/fonc.2021.790821 PMC8784869

[50] Toh CH, Siow TY, Castillo M (2021) Peritumoral brain edema in metastases may be related to glymphatic dysfunction. Front Oncol 11:725354. 10.3389/fonc.2021.725354 34722268 10.3389/fonc.2021.725354 PMC8548359

[51] Toh CH, Siow TY, Castillo M (2021) Peritumoral brain edema in meningiomas may be related to glymphatic dysfunction. Front Neurosci 15:674898. 10.3389/fnins.2021.674898 33967688 10.3389/fnins.2021.674898 PMC8100232

[52] Li L, Ding G, Zhang L et al (2023) Glymphatic transport is reduced in rats with spontaneous pituitary tumor. Front Med (Lausanne) 10:1189614. 10.3389/fmed.2023.1189614 37601793 10.3389/fmed.2023.1189614 PMC10436560

[53] Christensen J, Wright DK, Yamakawa GR, Shultz SR, Mychasiuk R (2020) Repetitive mild traumatic brain injury alters glymphatic clearance rates in limbic structures of adolescent female rats. Sci Rep 10(1):6254. 10.1038/s41598-020-63022-7 32277097 10.1038/s41598-020-63022-7 PMC7148360

[54] Li L, Chopp M, Ding G et al (2020) MRI detection of impairment of glymphatic function in rat after mild traumatic brain injury. Brain Res 1747:147062. 10.1016/j.brainres.2020.147062 32818526 10.1016/j.brainres.2020.147062 PMC9419050

[55] Iliff JJ, Chen MJ, Plog BA et al (2014) Impairment of glymphatic pathway function promotes tau pathology after traumatic brain injury. J Neurosci 34(49):16180–16193. 10.1523/jneurosci.3020-14.2014 25471560 10.1523/JNEUROSCI.3020-14.2014 PMC4252540

[56] Liu X, Xie Y, Wan X et al (2021) Protective effects of aquaporin-4 deficiency on longer-term neurological outcomes in a mouse model. Neurochem Res 46(6):1380–1389. 10.1007/s11064-021-03272-7 33651262 10.1007/s11064-021-03272-7

[57] Yang L, Chen Z, Wan X et al (2023) Angiotensin II type 1 receptor deficiency protects against the impairment of blood-brain barrier in a mouse model of traumatic brain injury. Int J Neurosci 133(6):604–611. 10.1080/00207454.2021.1946056 34219583 10.1080/00207454.2021.1946056

[58] Bolte AC, Dutta AB, Hurt ME et al (2020) Meningeal lymphatic dysfunction exacerbates traumatic brain injury pathogenesis. Nat Commun 11(1):4524. 10.1038/s41467-020-18113-4 32913280 10.1038/s41467-020-18113-4 PMC7483525

[59] Plog BA, Dashnaw ML, Hitomi E et al (2015) Biomarkers of traumatic injury are transported from brain to blood via the glymphatic system. J Neurosci 35(2):518–526. 10.1523/jneurosci.3742-14.2015 25589747 10.1523/JNEUROSCI.3742-14.2015 PMC4293408

[60] Opel RA, Christy A, Boespflug EL et al (2019) Effects of traumatic brain injury on sleep and enlarged perivascular spaces. J Cereb Blood Flow Metab 39(11):2258–2267. 10.1177/0271678x18791632 30092696 10.1177/0271678X18791632 PMC6827121

[61] Piantino J, Schwartz DL, Luther M et al (2021) Link between mild traumatic brain injury, poor sleep, and magnetic resonance imaging: visible perivascular spaces in veterans. J Neurotrauma 38(17):2391–2399. 10.1089/neu.2020.7447 33599176 10.1089/neu.2020.7447 PMC8390772

[62] Xiang T, Feng D, Zhang X et al (2022) Effects of increased intracranial pressure on cerebrospinal fluid influx, cerebral vascular hemodynamic indexes, and cerebrospinal fluid lymphatic efflux. J Cereb Blood Flow Metab 42(12):2287–2302. 10.1177/0271678x221119855 35962479 10.1177/0271678X221119855 PMC9670008

[63] Goyal N, Kumar P (2021) Putting ‘CSF-Shift Edema’ hypothesis to test: comparing cisternal and parenchymal pressures after basal cisternostomy for head injury. World Neurosurg 148:e252–e263. 10.1016/j.wneu.2020.12.133 33412318 10.1016/j.wneu.2020.12.133

[64] Bai Y, Yuan M, Mi H et al (2022) Hypothermia reduces glymphatic transportation in traumatic edematous brain assessed by intrathecal dynamic contrast-enhanced MRI. Front Neurol 13:957055. 10.3389/fneur.2022.957055 36341130 10.3389/fneur.2022.957055 PMC9632734

[65] Butler T, Zhou L, Ozsahin I et al (2023) Glymphatic clearance estimated using diffusion tensor imaging along perivascular spaces is reduced after traumatic brain injury and correlates with plasma neurofilament light, a biomarker of injury severity. Brain Comm 5(3):fcad134. 10.1093/braincomms/fcad134 10.1093/braincomms/fcad134 PMC10176239 37188222

[66] Hicks AJ, Sinclair B, Shultz SR et al (2023) Associations of enlarged perivascular spaces with brain lesions, brain age, and clinical outcomes in chronic traumatic brain injury. Neurology 101(1):e63–e73. 10.1212/wnl.0000000000207370 37156615 10.1212/WNL.0000000000207370 PMC10351302

[67] Liao J, Zhang M, Shi Z et al (2023) Improving the function of meningeal lymphatic vessels to promote brain edema absorption after traumatic brain injury. J Neurotrauma 40(3-4):383–394. 10.1089/neu.2022.0150 36106596 10.1089/neu.2022.0150

[68] Lv C, Han S, Sha Z et al (2023) Cerebral glucagon-like peptide-1 receptor activation alleviates traumatic brain injury by glymphatic system regulation in mice. CNS Neurosci Ther 29(12):3876–3888. 10.1111/cns.14308 37353947 10.1111/cns.14308 PMC10651945

[69] Liu M, Huang J, Liu T et al (2023) Exogenous interleukin 33 enhances the brain’s lymphatic drainage and toxic protein clearance in acute traumatic brain injury mice. Acta Neuropathol Commun 11(1):61. 10.1186/s40478-023-01555-4 37024941 10.1186/s40478-023-01555-4 PMC10080777

[70] Park JH, Bae YJ, Kim JS et al (2023) Glymphatic system evaluation using diffusion tensor imaging in patients with traumatic brain injury. Neuroradiology 65(3):551–557. 10.1007/s00234-022-03073-x 36274107 10.1007/s00234-022-03073-x

[71] Schwerin SC, Breehl N, Obasa A et al (2023) Actigraphic evidence of persistent sleep disruption following repetitive mild traumatic brain injury in a gyrencephalic model. Cereb Cortex 33(15):9263–9279. 10.1093/cercor/bhad199 37310176 10.1093/cercor/bhad199

[72] Yang DX, Sun Z, Yu MM et al (2023) Associations of MRI-derived glymphatic system impairment with global white matter damage and cognitive impairment in mild traumatic brain injury: a DTI-ALPS study. J Magn Reson Imaging. 10.1002/jmri.28797 10.1002/jmri.28797 37276070

[73] Dai Z, Yang Z, Li Z et al (2023) Increased glymphatic system activity in patients with mild traumatic brain injury. Front Neurol 14:1148878. 10.3389/fneur.2023.1148878 37251219 10.3389/fneur.2023.1148878 PMC10213560

[74] Chen JW, Xu JC, Malkasian D et al (2021) The mini-craniotomy for cSDH revisited: new perspectives. Front Neurol 12:660885. 10.3389/fneur.2021.660885 34025564 10.3389/fneur.2021.660885 PMC8134699

[75] Wolf S, Mielke D, Barner C et al (2023) Effectiveness of lumbar cerebrospinal fluid drain among patients with aneurysmal subarachnoid hemorrhage: a randomized clinical trial. JAMA Neurol 80(8):833–842. 10.1001/jamaneurol.2023.1792 37330974 10.1001/jamaneurol.2023.1792 PMC10277935

