# Supplementary material for: The glymphatic system for neurosurgeons: a scoping review
Source: Neurosurg Rev. 2024 Jan 23;47(1):61. doi: 10.1007/s10143-024-02291-6 (PMC10803566; doi:10.1007/s10143-024-02291-6)
Supplement: Supplementary file 1 — ESM 1 [file 10143_2024_2291_MOESM1_ESM.docx]

**Supplemental Table 1.** Studies investigating subarachnoid hemorrhage and the glymphatic system.

| **Paper (author and year)** | **Model for SAH (if applicable)** | **Species** | **Age** | **Number of subjects** | **Method for quantifying glymphatic function** | **Main results** | **Comments/other results** |
| --- | --- | --- | --- | --- | --- | --- | --- |
| Hou et al. [21] | Intracisternal blood infusion | Mouse | 8-12 weeks | N/A | Infusion of tracer substance in cisterna magna and evaluation at 6 hours, 1 day, 3 days and 7 days after SAH with fluorescence microscopy. | Reduced penetration of tracer substance in cortex and whole brain at 6 hours and 1 day after SAH compared to sham-mice. Reduced penetration of tracer substance in only cortex 3 and 7 days after SAH compared to sham-mice. | Prolonged increase of P-tau and decrease of AQP-4 expression was also seen in mice after SAH. Neuroinflammatory markers and ICP were also increased after SAH compared to sham-mice. All results together indicate decreased glymphatic function after SAH. |
| Pu et al. [23] | Intracisternal blood infusion | Mouse | 3 months | N/A | Intracisternal tracer injection followed by fluorescence microscopy of brain parenchyma, meningeal lymphatics and dCLN. | Higher accumulation of fluorescent tracer in meningeal lymphatic vessels and lower accumulation in dCLN. Reduced diffusion of tracer in the brain parenchyma after SAH in comparison with sham-mice. Increase in PVS size after SAH compared with sham-mice. | Increase in Tau and P-tau, brain water content, and depolarization of AQP-4 in astrocytic endfeet was also seen after SAH. All results together give evidence for decreased glymphatic function after SAH. |
| Liu et al. [22] | Perforation of ACA in circle of Willis in wild-type and AQP4 knockout (KO) rats | Rat | N/A | n=160 | Intracisternal and intrahippocampal Gd-DTPA injection followed by MRI and intracisternal and intrahippocampal fluorescent tracer followed by confocal fluorescence microscopy. | Decrease in parenchymal penetration of intracisternally injected Gd-DTPA in AQP4 KO rats. The decrease was aggravated by SAH. Decreased parenchymal spreading and clearance of intrahippocampally injected Gd-DTPA in AQP-4 KO rats with and without SAH. | Increase in brain water content and neurological deficits after SAH, aggravated by AQP4 KO. Decreased perivascular expression of AQP4 after SAH in wild-type mice. The results give evidence for impaired glymphatic function after SAH in rats. |
| Liu et al. [24] | Perforation of junction of middle and anterior cerebral arteries in wild-type and AQP4 knockout rats. | Rat | N/A | n=83 | Intracisternal Gd-DTPA injection followed by MRI. | Decrease in parenchymal penetration of Gd-DTPA in AQP4 knockout rats. SAH resulted in even longer parenchymal diffusion of tracer, and accumulation of tracer in the cisterna magna. | Significant increase in brain water content and decrease in neurological scoring after SAH in both wild-type and AQP4 KO rats. All results together indicate a negative effect of SAH on glymphatic function. |
| Liu et al. [27] | Perforation of intracerebral artery via suture advancement through the ICA in wild-type and AQP4 overexpressing mice. | Mouse | 8 weeks | N/A | Immunofluorescence microscopy of AQP4 (no objective quantification of glymphatic function) | AQP4 overexpression ameliorated AQP4 depolarization caused by SAH. | A decrease in ferroptosis and transferrin in the brain after SAH was also observed. Neurobehavioural improvement was observed in AQP4 overexpresssing mice. The decrease of depolarization could signify an improvement in glymphatic function in AQP-4 overexpressing mice. |
| Gaberel et al. [25] | Infusion of blood into pre-chiasmatic cistern | Mouse | N/A | N/A | Injection of DOTA-Gd in the cisterna magna followed by MRI. FITC-Dextran into cisterna magna followed by histological analysis. | SAH impaired DOTA-Gd parenchymal penetration. SAH resulted in accumulation of FITC-dextran tracer in the PVSs. | There was also evidence of fibrin and fibrinogen accumulation in the perivascular spaces, suggesting an involvement of perivascular clotting in the pathophysiology of impaired glymphatic function after SAH. |
| Golanov et al. [26] | Perforation of Circle of Willis | Mouse | 10-14 weeks | n=74 | Intracisternal gadopentetate injection followed by MRI and intracisternal Evans Blue, fluorescent tracers and microspheres with repeated subsequent visualization and quantification up to 30 days. | Tracer distribution completely blocked 4 days after SAH and severely impeded up to 30 days after SAH. | The use of fibrinogen antibodies showed that fibrin was present in paravascular spaces in areas far away from the SAH. Intraventricular injection of TF antibodies resulted in an increase in CSF-flow following SAH, indicating a role in fibrin deposition along the paravascular pathways in blocking CSF flow. Hemorrhage size was increased by the injection of TF antibodies. |
| Luo et al. [28] | Intracisternal arterial blood infusion (exception: one animal received laser puncture of pial arteriole). Hospitalized aneurysmal SAH patients. | Mouse and human | Mice: 8-12 weeks; Human: 19-68 years | Mice: n=65; Human: n=24 | Mice: No method for objective glymphatic function quantification, but quantification of fibrin deposition and clotting in paravascular spaces using various techniques; Human: No method for quantifying glymphatic function, but retrospective analysis of blood clots on CT angiography after SAH and H&E-staining of one SAH patient to identify paravascular erythrocyte presence. | Mice: Infiltration of subarachnoid blood into the paravascular spaces of the pial artery. Increase in ICP after SAH induction. Fibrinogen deposition was identified along the external wall of the paravascular spaces using the CLARITY-technique. Cerebral vasospasm was observed following SAH.; Human: Paravascular blood deposition was observed in the CT-angiography scans of SAH-patients. H&E-staining of one patient showed presence of erythrocyte presence along the paravascular spaces. | Increased expression of inflammatory markers and neurological decline was observed after SAH. Fasudil (Rho-kinase inhibitor) was not effective in improving neurological outcome or neuroinflammation. TPA proved helpful in improving the neurological outcome of SAH after 7 days. Deletion of AQP4 in mice did not significantly alleviate neurological decline nor ameliorate neuroinflammation after SAH. |
| Goulay et al. [29] | Optic cistern autologous blood injection | Monkey | Adult (age unspecified) | n=3 | Intracisternal DOTA-Gd injection followed by MRI | Parenchymal CSF-circulation was greatly impeded after SAH. | Immunohistochemistry showed fibrin presence between the astrocyte end feet and basal lamina of perivascular spaces, suggesting a role of occlusion of these spaces following SAH. |
| Kim et al. [30] | Patients with aneurysmal SAH | Human | 50.9 ± 13.1 years | Males: n=56; Females: n=83 | Quantification of enlarged PVS in centrum semiovale and basal ganglia on T2-weighted MRI. | 99 patients were available for follow-up MRI. Significant correlation between the size of SAH and enlarged PVS aggravation in centrum semiovale. | There was also a correlation between initial enlarged PVS in CSO and the aggravation of enlarged PVS on the follow-up MRI. This indicates a predictive role of the initial enlarged PVS. |
| Hou et al. [31] | Intracisternal autologous blood infusion | Mouse | 8-12 weeks | n=123 (n=102 included in the end) | Intracisternal fluorescent tracer injection | Parenchymal penetration of CSF-tracer was significantly reduced after SAH. Reduction of parenchymal penetration of CSF-tracer was partly reversed by nimodipine treatment. | Increase in P-tau/tau-ratio and AQP4 depolarization observed after SAH, both attenuated after treatment with nimodipine. Cortical blood flow and neurological outcome in the mice was also improved with nimodipine treatment after SAH. |
| Fang et al. [32] | Perforation of junction of middle and anterior cerebral arteries. | Rat | Adult (age unspecified) | n=334 | Intracisternal Evans blue administration and subsequent macroscopic quantification of the distribution of the tracer. | Exogenous PACAP mediated an increase in glymphatic system function compares with administration of PBS in rats with SAH. A decrease in ICP, brain water content and neurological deficits was seen after treatment with exogenous PACAP of rats with SAH. | Exogenous PACAP caused an attenuation of post-SAH glymphatic dysfunction through attenuating SUR1-mediated depolarization of AQP4 channels in astrocytes, giving evidence for the role of AQP4 in glymphatic function. |
| Liu et al. [33] | Perforation of anterior cerebral artery in AQP4-deficient rats (knockout) | Rat | 280–300 g, age unspecified | N=160 | MRI, fluorescent imaging, and transmission electron  microscopy (TEM) | Level of AQP4 was increased around the arteries after SAH, the level of  AQP4 around the veins did not change markedly. Clearance rate in the interstitial space was significantly decreased after SAH, especially in the AQP4 knockout group. |  |

AQP4 = aquaporin-4; CSF = cerebrospinal fluid; dCLN = draining cervical lymph nodes; FITC-Dextran = fluorescein isothiocyanate-Dextran; Gd; DTPA = Gadopentetic acid; H&E = Hematoxylin and eosin (stain); ICP = intracranial pressure; PACAP = Pituitary Adenylate Cyclase-Activating Polypeptide; P-tau = phosphorylated tau; PVS = perivascular space; SAH = subarachnoid hemorrhage; TF = tissue factor (brain coagulation factor III)
