# Supplementary material for: The glymphatic system for neurosurgeons: a scoping review
Source: Neurosurg Rev. 2024 Jan 23;47(1):61. doi: 10.1007/s10143-024-02291-6 (PMC10803566; doi:10.1007/s10143-024-02291-6)
Supplement: Supplementary file 2 — ESM 2 [file 10143_2024_2291_MOESM2_ESM.docx]

**Supplemental Table 2.** Studies investigating stroke and the glymphatic system.

| **Paper (author and year)** | **Model for stroke (if applicable)** | **Species** | **Age** | **Number of subjects** | **Method for quantifying glymphatic function** | **Main results** | **Comments/other results** |
| --- | --- | --- | --- | --- | --- | --- | --- |
| Gaberel et al. [25] | MCAO (Middle cerebral artery occlusion) | Mouse | N/A | N/A | Intracisternal Gd-DTPA injection followed by repeated MRI imaging and intracisternal tracer injection followed by fluoresence microscopy. | Parenchymal penetration of both Gd-DTPA and fluorescent tracer was severely impaired 3 hours after MCAO. | The impairment persisted even after bilateral craniotomy, ruling out intracranial hypertension as the main cause of glymphatic dysfunction. |
| Zhang et al. [34] | Spontaneous intracerebral hemorrhage (sICH) patients | Human | Patients: 47.8 ± 12.4 years; Control subjects: 47.3 ± 10.9 years | Patients: n=20; Control subjects: n=31 | Calculation of ALPS-index using DTI-ALPS. | ALPS-index was significantly decreased in sICH patients, both when comparing to healthy control and comparing the lesion side to the contralateral side in the same patient. |  |
| Mestre et al. [35] | MCAO | Mouse | 8 weeks | N/A | Intracisternal fluorescent tracer and gadobutrol injection followed by microscopic/macroscopic analysis and MRI, respectively. | Influx of CSF-tracer into the brain parenchyma from the lateral ventricles, cisterna magna and the paravascular spaces after MCAO. Enrichment of gadobutrol ipsilaterally in paravascular spaces following MCAO. PVS size was increased and PVS pressure was lowered following MCAO, indicating vasoconstriction. | Other experiments in the study suggested that ischemic spreading depolarization following the ischemic stroke caused a vasoconstriction of penetrating and pial arterioles and concurrent enrichment of intracisternally injected tracer substance in the parenchyma. AQP4 knockout in mice resulted in a reduction of brain edema and intracisternally injected tracer substance in the brain parenchyma following MCAO in mice. All results taken together indicate a role of glymphatic dysfunction in the formation of ischemic brain edema following ischemic stroke. |
| Sun et al. [36] | MCAO | Rat | Adult (age unspecified) | n=16 | No objective measurement of glymphatic function was performed. | T2-WI showed that the volume of both the ischemic lesion and edema were reduced in rats treated with TGN-020 (AQP4 inhibitor) in comparison with control rats after MCAO. | Neurological decline and astrogliosis was also reduced after treatment with TGN-020 in comparison with untreated rats. AQP4 depolarization was also lower in TGN-020-treated rats. |
| Lin et al. [37] | MCAO | Rat | Adult (age unspecified) | n=12 | Intracisternal Gd-DTPA injection followed by repeated MRI imaging. | Both inflow and clearance of Gd-DTPA was slowed in the ipsilateral substantia nigra compared to the contralateral in the subacute phase (7 days after MCAO). Slower clearance of Gd-DTPA from the ipsilateral ventral thalamic nucleus was seen in comparison to the contralateral side in the subacute phase (7 days after MCAO). | No significant difference in Gd-DTPA signal intensity was seen at the acute phase (1 day after MCAO) in either of the ROIs (substantia nigra and ventral thalamic nucleus). |
| Toh and Siow [38] | Patients with ischemic stroke | Human | Patients: 56.7 ± 15.2 years; Control subjects: 53.3 ± 9.9 years | Patients: n=50; Normal subjects: n=44 | Calculation of ALPS-index using DTI-ALPS. | ALPS-index was significantly lower in the ipsilateral hemisphere to the infarct in comparison with the contralateral hemisphere. ALPS index was also lower in the ipsilateral hemisphere in ischemic stroke patients in comparison with control subjects. Smaller stroke lesion size was also associated with higher ALPS index after longer time post-stroke, indicating glymphatic recovery in these patients. | ALPS index also correlated negatively with stroke volume. All these results taken together indicate that ischemic stroke results in glymphatic dysfunction, as the ALPS index is a biomarker for fluid diffusion along the perivascular routes. |
| Tsai et al. [39] | Spontaneous intracerebral hemorrhage (sICH) patients with cerebral amyloid angiopathy (CAA-ICH) and without cerebral amyloid angiopathy (Non-CAA-ICH). | Human | CAA-ICH: 72.4 ± 12.0 years; non-CAA-ICH: 61.3 ± 11.2 years | CAA-ICH: n=29; non-CAA-ICH: n=79 | Visibility of perivascular spaces in centrum semiovale and basal ganglia in MRI scans. | No significant difference was found between the CAA-ICH and non-CAA-ICH groups in terms of visibility of centrum semiovale and basal ganglia perivascular spaces. | There was a significant correlation between high PiB retention (11C-Pittsburgh compound B, a measure of amyloid deposition) and radiological PVS visibility, suggesting a connection between glymphatic dysfunction and cerebral amyloid deposition. |
| Liu et al. [40] | Intracerebral hemorrhage (ICH) was experimentally induced. | Rat | 8-10 weeks | n=75 | No objective measurement of glymphatic function was performed. | Ligation and removal of lymph nodes (cerebral lymphatic blockage, CLB) resulted in a lack of recovery of cerebral edema after ICH induction in comparison with ICH-only rats. AQP4 expression was significantly lower in CLB-ICH rats in comparison with ICH-only rats. | Neuroinflammation, neurological performance and neuronal apoptosis was also worsened by CLB-ICH in comparison with ICH-only rats. All results taken together could give evidence for the importance of glymphatic and lymphatic drainage systems for cerebral recovery after ICH. |
| Shanbhag et al. [41] | Idiopathic subdural hematoma (SDH) | Pig | N/A | n=1 | Intracisternal fluorescent tracer injection followed by macroscopic fluorescence analysis. | Impairment of CSF tracer distribution in the whole brain after subdural hematoma. | The impaired tracer distribution was hypothesized to be mediated by increased ICP caused by the SDH. |
| Zhang et al. [42] | MCAO (Middle cerebral artery occlusion) | Mouse | 10-12 weeks | N/A | Intracisternal BOPTA-Gd injection | Reduction in signal intensity of contrast agent in the cortex, striatum and both cerebral ventricles after MCAO in comparison with sham-mice in all ROIs. T1W1 showed a positive correlation between ADC (apparent diffusion coefficient) and contrast signal intensity in all ROIs, indicating a correlation between brain edema and glymphatic dysfunction. | Immunohistochemical staining of AQP4 revealed an impairment of perivascular AQP4 polarity and a decrease in AQP4 expression along the eppendymal wall. These result further strenghten the theory of glymphatic impairment resulting from ischemic stroke. |
| Yi et al. [43] | Stroke induced by temporary middle cerebral artery occlusion and reperfusion surgery. | Mouse | 8-12 weeks | n=108 | Fluorescent tracers injected into cisterna magna, studied by immunofluorescence. | Pretreatment with Xuefu Zhuyu (traditional Chinese herb) improved the neurological score in mice, in addition to enchance CSF fluid influx and protected AQP4 depolarization. | The authors suggest Xuefu Zhuyu shows promising results in reducing the onset and progression of ischemic stroke. |
| Qin et al. [44] | Subacute ischemic stroke | Human |  | Ischemic stroke n=20, healthy controls n=30 | DTI-ALPS | Lower DTI-ALPS index was found in the group with ischemic stroke compared to healthy controls. | Glymphatic system seems to be involved in subacute ischemic stroke. |
| Li et al. [45] | Ischemic stroke after MCAO | Rat | 260-270 g, age not mentioned | n=20 | DCE-MRI and immunofluorescence staining | At 2nd week after MCAO the glymphatic system in thalamus was impaired. |  |
| Liu et al. [46] | Intracerebral hemorrhage (ICH) was experimentally induced. | Mouse | 7-9 weeks | n=442 | Injection tracer in cisterna magna, then brains were examined using a fluorescence microscope. | ICH group showed impaired glymphatic function compared to sham group, and transcranial magnetic stimulation was shown to improve the clearance of CSF tracers. |  |

MCA = DTI-ALPS = Diffusion tensor imaging along the perivascular space; BOPTA-Gd = gadobenate dimeglumine (contrast agent); CAA-ICH = intracerebral hemorrhage with cerebral amyloid angiopathy; CLB = cerebral lymphatic blockage; CSF = cerebrospinal fluid; DCE-MRI = Dynamic Contrast-Enhanced Magnetic Resonance Imaging; Gd-DTPA = Gadopentetic acid; ICH = intracerebal hemorrhage; non-CAA-ICH = intracerebral hemorrhage without cerebral amyloid angiopathy; PVS = perivascular space; ROIs = regions of interest; T1W1 = T1-Weighted Imaging; TGN-020 = aquaporin-4 inhibitor
