# Supplementary material for: The glymphatic system for neurosurgeons: a scoping review
Source: Neurosurg Rev. 2024 Jan 23;47(1):61. doi: 10.1007/s10143-024-02291-6 (PMC10803566; doi:10.1007/s10143-024-02291-6)
Supplement: Supplementary file 3 — ESM 3 [file 10143_2024_2291_MOESM3_ESM.docx]

**Supplemental Table 3.** Studies investigating traumatic brain injury and the glymphatic system.

| **Paper (author and year)** | **Main intervention (if applicable)** | **Species** | **Age** | **Number of subjects** | **Main method for quantifying glymphatic function (when applicable)** | **Main results** | **Comments/other results** |
| --- | --- | --- | --- | --- | --- | --- | --- |
| Christensen et al. [53] | Repetitive impact mild traumatic brain injury (RmTBI) | Rat | 3 mTBIs at P30, P34 and P38 respectively | n=11 (RmTBI: n=6, sham: n=5) | Intracisternal Gadovist infusion and MRI | Increased influx of contrast agent to several brain areas, with consequent decrease in efflux in the same areas in rats with RmTBI. Impeded behavioral and neurological parameters in rats with RmTBI. | Areas that were affected included the amygdala, hypothalamus, hippocamppus and olfactory bulb. Decreased contrast agent efflux is hypothesized to be caused by decreased glymphatic clearance. Increased glymphatic influx combined with decreased glymphatic efflux is hypothesized to play a part in the pathogenesis of brain edema. |
| Li et al. [54] | Single impact mTBI | Rat | N/A | n=14 (mTBI: n=7, sham: n=7) | Intracisternal Gd-DTPA infusion and MRI | Decreased influx and efflux of contrast agent in several brain areas after mTBI in comparison with sham mice. | Areas that were affected included the olfactory bulb, cortex, hippocampus, thalamus, hypothalamus and cerebellum (the olfactory bulb was affected the most). |
| Iliff et al. [55] | Single impact mTBI with and without AQP4 knockout. | Mouse | 8-12 weeks | N/A | Intracisternal injection of fluorescent tracer, and AQP4 and P-tau protein quantification (immunohistochemistry) | Impairment of tracer penetration, decrease in paravascular AQP4 polarization and higher accumulation of P-tau after mTBI. Impairments were more pronounced in AQP4 knockout mice. | Glial scarring and cognitive impairment were present after mTBI, and was also more pronounced in AQP4 knockout mice. |
| Liu et al. [56] | Craniotomy followed by single impact mTBI toward cortical surface. | Mouse | 8-12 weeks | n=160 | Comparison between AQP4 knockout mice and wild type-mice and quantification of amyloid beta protein levels (western blot) and brain water content. | Significant increase in brain water content and amyloid beta after mTBI. Both brain water content increase (edema) and amyloid beta protein increase were ameliorated by AQP4 knockout. | No objective quantification of glymphatic function was done, although this study aims to give evidence for neuroprotective effects of AQP4 knockout in mice subjected to mTBI. Increase in inflammatory markers after mTBI was lower in AQP4 knockout mice. Blood brain barrier-integrity was also higher in AQP4 knockout mice. |
| Yang et al. [57] | Craniotomy followed by single impact mTBI toward cortical surface with and without AT1a receptor knockout. | Mouse | 3 months | n=128 | Measurement of AQP4 polarization on astrocytic end feet and cell bodies (immunohistochemistry). Measurement of brain water content. Measurement of clearance of amyloid-beta protein (western blotting). | mTBI resulted in loss of AQP4 polarization, reduced clearance of amyloid beta protein, and high brain water content (edema) in wild type-mice. All of the aforementioned were less pronounced in AT1a-receptor knockout mice. | No objective quantification of glymphatic function was done. Decreased blood brain barrier integrity was seen after mTBI in wild type-mice, and to a lesser degree in AT1a knockout mice. |
| Bolte et al. [58] | Single impact mTBI toward closed skull with and without bilateral ligation of the internal jugular veins. | Mouse | 8-10 weeks | N/A | Intracisternal injection of 0.5 µm fluorescent beads followed by quantification of fluorescent beads in dCLN, meninges and brain. Measurement of ICP. Confocal microscopy of meningeal lymphatic vessels. No objective assessment of glymphatic function was done. | Reduced meningeal lymphatic drainage from 2 hours up to 1 month after after mTBI. Altered meningeal lymphatic morphology was observed after mTBI. Bilateral ligation of internal jugular veins resulted in increased ICP and impairment of meningeal lymphatic drainage. | No real quantification of glymphpatic function was done, as the beads used are too large to go through the glymphatic system. Only an assessment of meningeal lymphatics was done in this study. |
| Plog et al. [59] | Single impact mTBI toward closed skull + manipulation of glymphatic clearance using one of the following: 1) AQP4 knockout; 2) cisternotomy; 3) acetazolamide treatment; 4) sleep deprivation | Mouse | 8-12 weeks | N/A | Intracortical fluorescent protein injection and microscopy of deep cervical lymph nodes (in vivo epifluorescence microscopy and ex vivo fluorescence imaging). Intracortical injection of radioisotope tracer substances (3H-dextran and 14C-inulin) followed by ex vivo analysis. Analysis of glymphatic clearance of TBI biomarkers to the blood (S100beta, GFAP and NSE). | All glymphatic system manipulations resulted in a decrease of tracer substances reaching the deep cervical lymph nodes. All glymphatic system manipulations resulted in a decrease in TBI biomarker presence in blood. | Although no objective measurement of glymphatic clearance was done, clearance of intracortically injected tracers and presence of TBI biomarkers in blood were used as surrogate markers. Analysis of blood brain barrier permeability (intravenous Evans blue) also showed no significant change after glymphatic clearance manipulation. |
| Opel et al. [60] | Subjected to TBI in the past. | Human | 57.1 ± 14.1 years (No TBI: 59.8 ± 12.3; TBI: 52.0 ± 16.5) | n=38 (n=25 for "No TBI" and n=13 for "TBI") | Manual quantification of enlarged perivascular spaces (ePVS) via MRI imaging. | High ePVS count positively correlated with shorter total sleep time and lower percentage of REM sleep quantified with polysomnography. | The results may point to a correlation between glymphatic dysfunction and decreased sleep quality. |
| Piantino et al. [61] | War veterans with mTBI. | Human | Median = 32 (29-41) | n=56 | Automated segmentation, detection and quantification of PVS burden, later controlled manually. | High PVS burden was positively correlated with the amount of mTBIs, systolic blood pressure, persistence of poor balance. | High PVS burden indicate dilation of the perivascular spaces, which would suggest impaired clearance of metabolic waste via the glymphatic pathways. |
| Xiang et al. [62] | Increased intracranial pressure induced by inflating balloon model. | Mouse and rat | Mice: 8-10 weeks, rats: 9-10 weeks. | N/A | Injection of fluorescent tracers into the cisterna magna. | Significant impairment of the glymphatic circulation after induced increased ICP as well as interruption of the drainage of deep cervical lymph nodes. | Increased ICP significantly impacts the glymphatic-lymphatic fluid transport system. |
| Goyal and Kumar [63] | Patients with TBI | Human | Median: 45 (25-72) | n=9 | No objective measurement of glymphatic function was performed. | Cisternal pressure reduction resulting from complete or partial basal cisternotomy in combination with decompressive hemicraniotomy was seen together with clinical improvement and alleviation of brain edema, whereas an increase in cisternal pressure after surgery was associated with clinical exacerbation. | This study tested the "CSF-shift edema" hypothesis, in which it is postulated that an increase in intracisternal pressure causes a shift of the pressure gradient between the intracisternal pressure and intraparenchymal pressure, resulting in brain edema. No actual statistical analyses were performed in this study, as the patients were too few. |
| Bai et al. [64] | TBI with hypothermia treatment | Rat | 3 months | n=28 | Dynamic contrast-enhanced MRI (DCE-MRI) after injection of contrast agent in cisterna magna performed after the TBI and hypothermia. | Hypothermia attenuated brain edema. Hypothermia caused worsening of glymphatic drainage. They also found lower glymphatic transport and vasogenic edema to be related to lower AQP4-expression. |  |
| Butler et al. [65] | TBI | Human | TBI 51.5 years (SD 16.7), non-TBI controls 58.5 years (SD 15.1) | TBI n=37, non-TBI controls n=13. | DTI-ALPS | ALPS index was lower in subjects after TBI compared with controls. The index was also negatively correlated with levels of neurofilament light chain in plasma. | DTI-ALPS seems to be a promising method for estimating glymphatic clearance in TBI patients. |
| Hicks et al. [66] | TBI | Human | TBI 56.8 years, non-TBI controls 59.8 years | TBI n=100, non-TBI controls n=75 | Evaluation of enlarged perivascular spaces (ePVS) using with 3T MRI, automated segmentation. | TBI was associated with greater ePVS and associated with reduced verbal memory performance. | ePVS may indicate glymphatic system impairments in patients after TBI in the chronic period after injury. |
| Liao et al. [67] | Induced TBI. Evaluated effect of ketoprofen, 9-cisRA and VEGF-C on meningeal lymphatic vessels. | Rat | 270-300g | Not stated | Contrast injected in cerebellomedullary cistern, 30 minutes later the cervical lymph nodes and brain tissue were collected. Additionally, 3T MRI was used. | The authors found ketoprofen, 9-cisRA and VEGF-C can improve function of meningeal lymphatic vessels | May be used in treatment of brain edema after TBI. |
| Lv et al. [68] | Glymphatic system regulation by cerebral glucagon-like peptide 1 receptor (GLP-1R) activation after TBI. | Mouse | 8-12 weeks | n=124 | Animals divided into four experiments. Tracer injected into cisterna magna. Immunofluorescence used for AQP4 polarization evaluation. | Activation of GLP-1R restored glymphatic transport after TBI and can improve pathological changes after TBI. | Could be used to repair glymphatic system damage caused by TBI. |
| Liu et al. [69] | Examination of interleukin 33 (IL-33) effect on glymphatic system after TBI. | Mouse | 8-10 weeks | n=24-32, not completely clear | CSF tracer injected in cisterna magna | When administrated IL-33 the TBI mice showed improved motor and memory skills. It also increased exchange of CSF and interstitial fluid, reversed dysregulation and depolarization of AQP4 which improved drainage to deep cervical lymph nodes. | IL-33 shows potential to be used in treatment after TBI. |
| Park et al. [70] | Evaluation of glymphatic system in patients with TBI | Human | TBI median age 36 years (20-50) and non-TBI controls 32 years (25-49) | TBI n=89, non-TBI controls n=34 | DTI-ALPS | ALPS index was lower in TBI patients. Presence of subarachnoid hemorrhage and diffuse axonal injury were correlated to lower ALPS index. | DTI-ALPS methos can be useful for evaluating impairment of glymphatic system. |
| Schwerin et al. [71] | Evaluation of sleep disruption after mTBI in gyrencephalic model. | Ferret | 33-90 weeks | n=71 | Immunohistochemistry | GFAP was greater in injury groups compared to sham but did not differ 6 months after trauma. The intensity of AQP4 differed between TBI and control group, even after 6 months in group with TBI and sleep disruption, suggesting key role in glymphatic system. |  |
| Yang et al. [72] | Assessment of glymphatic function after mTBI by studying perivascular space | Human | mTBI 48.7 years (SD 14.1), non-TBI controls 49.2 years (SD 14.0) | mTBI n=58, non-TBI controls n=34 | DTI-ALPS | Lower DTI-ALPS was seen in complicated TBI. Glymphatic function may be impaired in mTBI. | Cognitive impairment can be caused by glymphatic dysfunction related to global white matter damage after mTBI. |
| Dai et al. [73] | mTBI | Human | TBI 48.9 years (SD 20.2), non-TBI controls 55.6 years (SD 17.7) | TBI n=161, non-TBI controls n=28 | DTI-ALPS | Based on ALPS index, the glymphatic activity was increased in mTBI patients, even with negative MRI. |  |
| Chen et al. [74] | Mini-craniotomy after cSDH (chronic subdural hematomas) | Human | Mean age: 68.9 ± 19.7 years | n=34 | No quantification made | No recurrence of symptoms or deaths after surgery. Better chance at decompression via fenestration of inner membrane of cSDH in comparison to burr holes. | Mini-Craniotomy may be a good surgical method for treating cSDH, but a larger sample size and longer follow-up is needed to carry out a statistical analysis of surgical outcome. It is hypothesized that this technique may enhance glymphatic flow postoperatively. |

AQP4 = aquaporin-4; AT1a = Angiotensin II type 1; dCLN = draining cervical lymph nodes; DTI-ALPS = Diffusion tensor imaging along the perivascular space; ePVS = enlarged perivascular spaces; Gd-DTPA = Gadopentetic acid (contrast agent for MRI); GLP-1R = cerebral glucagon-like peptide 1 receptor ; ICP = intracranial pressure; IL-33 = Interleukin-33; MRI = magnetic resonance imaging; mTBI = mild traumatic brain injury; P-tau = phosphorylated tau; PVS burden = burden or load of perivascular spaces visible on MRI; RmTBI = Repetitive impact mild traumatic brain injury

**References in Supplemental Table 3**

52. Christensen J, Wright DK, Yamakawa GR, Shultz SR, Mychasiuk R. Repetitive Mild Traumatic Brain Injury Alters Glymphatic Clearance Rates in Limbic Structures of Adolescent Female Rats. Scientific reports. Apr 10 2020;10(1):6254. doi:10.1038/s41598-020-63022-7

53. Li L, Chopp M, Ding G, et al. MRI detection of impairment of glymphatic function in rat after mild traumatic brain injury. Brain Res. Nov 15 2020;1747:147062. doi:10.1016/j.brainres.2020.147062

54. Iliff JJ, Chen MJ, Plog BA, et al. Impairment of glymphatic pathway function promotes tau pathology after traumatic brain injury. J Neurosci. Dec 3 2014;34(49):16180-93. doi:10.1523/jneurosci.3020-14.2014

55. Liu X, Xie Y, Wan X, Wu J, Fan Z, Yang L. Protective Effects of Aquaporin-4 Deficiency on Longer-term Neurological Outcomes in a Mouse Model. Neurochem Res. Jun 2021;46(6):1380-1389. doi:10.1007/s11064-021-03272-7

56. Yang L, Chen Z, Wan X, et al. Angiotensin II type 1 receptor deficiency protects against the impairment of blood-brain barrier in a mouse model of traumatic brain injury. The International journal of neuroscience. Jun 2023;133(6):604-611. doi:10.1080/00207454.2021.1946056

57. Bolte AC, Dutta AB, Hurt ME, et al. Meningeal lymphatic dysfunction exacerbates traumatic brain injury pathogenesis. Nature Communications. 2020/09/10 2020;11(1):4524. doi:10.1038/s41467-020-18113-4

58. Plog BA, Dashnaw ML, Hitomi E, et al. Biomarkers of traumatic injury are transported from brain to blood via the glymphatic system. J Neurosci. Jan 14 2015;35(2):518-26. doi:10.1523/jneurosci.3742-14.2015

59. Opel RA, Christy A, Boespflug EL, et al. Effects of traumatic brain injury on sleep and enlarged perivascular spaces. J Cereb Blood Flow Metab. Nov 2019;39(11):2258-2267. doi:10.1177/0271678x18791632

60. Piantino J, Schwartz DL, Luther M, et al. Link between Mild Traumatic Brain Injury, Poor Sleep, and Magnetic Resonance Imaging: Visible Perivascular Spaces in Veterans. J Neurotrauma. Sep 1 2021;38(17):2391-2399. doi:10.1089/neu.2020.7447

61. Xiang T, Feng D, Zhang X, et al. Effects of increased intracranial pressure on cerebrospinal fluid influx, cerebral vascular hemodynamic indexes, and cerebrospinal fluid lymphatic efflux. J Cereb Blood Flow Metab. Dec 2022;42(12):2287-2302. doi:10.1177/0271678x221119855

62. Goyal N, Kumar P. Putting 'CSF-Shift Edema' Hypothesis to Test: Comparing Cisternal and Parenchymal Pressures After Basal Cisternostomy for Head Injury. World neurosurgery. Apr 2021;148:e252-e263. doi:10.1016/j.wneu.2020.12.133

63. Bai Y, Yuan M, Mi H, et al. Hypothermia reduces glymphatic transportation in traumatic edematous brain assessed by intrathecal dynamic contrast-enhanced MRI. Front Neurol. 2022;13:957055. doi:10.3389/fneur.2022.957055

64. Butler T, Zhou L, Ozsahin I, et al. Glymphatic clearance estimated using diffusion tensor imaging along perivascular spaces is reduced after traumatic brain injury and correlates with plasma neurofilament light, a biomarker of injury severity. Brain Commun. 2023;5(3):fcad134. doi:10.1093/braincomms/fcad134

65. Hicks AJ, Sinclair B, Shultz SR, et al. Associations of Enlarged Perivascular Spaces With Brain Lesions, Brain Age, and Clinical Outcomes in Chronic Traumatic Brain Injury. Neurology. Jul 4 2023;101(1):e63-e73. doi:10.1212/wnl.0000000000207370

66. Liao J, Zhang M, Shi Z, et al. Improving the Function of Meningeal Lymphatic Vessels to Promote Brain Edema Absorption after Traumatic Brain Injury. J Neurotrauma. Feb 2023;40(3-4):383-394. doi:10.1089/neu.2022.0150

67. Lv C, Han S, Sha Z, et al. Cerebral glucagon-like peptide-1 receptor activation alleviates traumatic brain injury by glymphatic system regulation in mice. CNS Neurosci Ther. Jun 23 2023;doi:10.1111/cns.14308

68. Liu M, Huang J, Liu T, et al. Exogenous interleukin 33 enhances the brain's lymphatic drainage and toxic protein clearance in acute traumatic brain injury mice. Acta Neuropathol Commun. Apr 7 2023;11(1):61. doi:10.1186/s40478-023-01555-4

69. Park JH, Bae YJ, Kim JS, et al. Glymphatic system evaluation using diffusion tensor imaging in patients with traumatic brain injury. Neuroradiology. Mar 2023;65(3):551-557. doi:10.1007/s00234-022-03073-x

70. Schwerin SC, Breehl N, Obasa A, et al. Actigraphic evidence of persistent sleep disruption following repetitive mild traumatic brain injury in a gyrencephalic model. Cerebral Cortex. 2023;33(15):9263-9279. doi:10.1093/cercor/bhad199

71. Yang DX, Sun Z, Yu MM, et al. Associations of MRI-Derived Glymphatic System Impairment With Global White Matter Damage and Cognitive Impairment in Mild Traumatic Brain Injury: A DTI-ALPS Study. J Magn Reson Imaging. Jun 5 2023;doi:10.1002/jmri.28797

72. Dai Z, Yang Z, Li Z, et al. Increased glymphatic system activity in patients with mild traumatic brain injury. Front Neurol. 2023;14:1148878. doi:10.3389/fneur.2023.1148878

73. Chen JW, Xu JC, Malkasian D, Perez-Rosendahl MA, Tran DK. The Mini-Craniotomy for cSDH Revisited: New Perspectives. Front Neurol. 2021;12:660885. doi:10.3389/fneur.2021.660885
